# Radical‐Mediated, Substrate‐Independent Fabrication of Hybrid Solid–Hydrogel Materials With Tunable Crosslinking: An Initiator‐ and Crosslinker‐Free Approach

**DOI:** 10.1002/advs.202516300

**Published:** 2026-01-21

**Authors:** Ghazal Shineh, Azin Khodaei, Pardis Keikhosravani, Masoud Zhianmanesh, Yiyun Xia, Aaron Gilmour, Sina Naficy, Andrea Guzina, Daniella Bianchi, Khoon S. Lim, Fariba Dehghani, Saber Amin Yavari, Marcela Bilek, Giselle C. Yeo, Behnam Akhavan

**Affiliations:** ^1^ School of Biomedical Engineering University of Sydney Sydney New South Wales 2006 Australia; ^2^ School of Engineering University of Newcastle Callaghan New South Wales 2308 Australia; ^3^ Charles Perkins Centre University of Sydney Sydney New South Wales 2006 Australia; ^4^ Department of Orthopedics University Medical Center Utrecht Utrecht 3508GA The Netherlands; ^5^ School of Chemical and Biomolecular Engineering The University of Sydney Darlington New South Wales 2008 Australia; ^6^ School of Medical Sciences The University of Sydney Darlington New South Wales 2008 Australia; ^7^ Regenerative Medicine Utrecht Utrecht University Utrecht The Netherlands; ^8^ The University of Sydney Nano Institute University of Sydney Sydney New South Wales 2006 Australia; ^9^ School of Physics University of Sydney Sydney New South Wales 2006 Australia; ^10^ School of Life and Environmental Sciences University of Sydney Sydney New South Wales 2006 Australia; ^11^ Hunter Medical Research Institute (HMRI) New Lambton Heights Newcastle New South Wales 2305 Australia

**Keywords:** covalent bonding, hydrogel–solid hybrids, plasma polymerization, radical‐mediated crosslinking, soft tissue integration

## Abstract

Achieving robust, cytocompatible bonding of hydrogels to solid substrates remains a long‐lasting challenge in the development of hybrid solid–hydrogel (HSH) systems for biomedical applications. Current strategies for hydrogel–solid bonding suffer from the complexity of processes, toxicity from residual crosslinkers, and substrate dependency; issues that hinder clinical adoption of HSH structures (HSHs). Overcoming these impediments, a dry, reagent‐free strategy is presented to create radical‐rich interlayers that enable initiator‐ and crosslinker‐free covalent attachment of hydrogels for the fabrication of robust HSHs. Evidence is provided in which long‐lived radicals embedded in ion‐assisted plasma polymerized coatings simultaneously drive hydrogel anchoring and in situ crosslinking on diverse non‐polymeric substrates, including titanium, stainless steel, and glass. GelMA, chitosan, and PVA‐Tyr hydrogels are immobilized with high stability, with coatings remaining intact after two months in aqueous media. Tuning the substrate bias voltage modulates radical concentration, enabling precise control over hydrogel thickness and crosslinking density with no need for extra reagents and/or crosslinkers. Cytocompatibility is confirmed with human mesenchymal stem cells and macrophages, with negligible inflammatory activation detected under the tested conditions. To showcase one application among many, fibroblasts on GelMA‐based HSHs exhibited enhanced early attachment, spreading, and proliferation, supporting their application in promoting soft tissue integration. This substrate‐independent, additive‐ and initiator‐free strategy embodies high‐quality‐by‐design principles, enabling a universal and scalable platform for the fabrication of HSH systems, particularly suited for applications requiring seamless integration between soft and hard materials, such as biomedical coatings, tissue‐interfacing constructs, and next‐generation soft robotics.

## Graphical abstract

This work introduces a substrate‐independent, reagent‐free plasma strategy that forms radical‐rich interlayers for covalent hydrogel attachment without initiators or crosslinkers. The long‐lived radicals drive in situ gelation, creating robust, cytocompatible hybrid solid–hydrogel constructs across diverse substrates. The approach enables tunable interfaces and offers a scalable pathway for tissue‐interfacing systems and emerging soft‐robotic technologies.

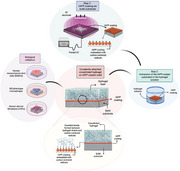

## 1 Introduction

Hydrogels, 3D polymeric networks swollen with water, exhibit distinct physicochemical properties, including low surface sliding friction, high permeability, and pronounced antifouling behavior.^[^ ^1^, ^2^, ^3^, ^4^, ^5^, ^6^ ^]^ Their intrinsic water content, combined with broad fabrication versatility, has enabled their deployment across diverse application spaces; from tissue engineering constructs ^[^ ^7^, ^8^, ^9^, ^10^, ^11^, ^12^ ^]^ and optical actuators ^[^ ^13^ ^]^ to marine antifouling coatings ^[^ ^14^ ^]^ and soft robotic systems.^[^ ^15^, ^16^ ^]^ Their extracellular matrix (ECM)‐mimetic nature holds further promise for promoting tissue integration in engineered systems by supporting cellular adhesion and proliferation.^[^ ^17^, ^18^ ^]^

Despite these advantages, two long‐standing challenges have limited the large‐scale application of hydrogels in engineered systems: their inherently weak mechanical properties and their inability to conform to mechanically robust or anatomically complex substrates, for example a meniscus in a human joint, or a complex, flexible circuit board.^[^ ^19^ ^]^ A paradigm‐shifting solution emerges from the integration of hydrogels with solid materials, giving rise to hybrid solid–hydrogel (HSH) structures that overcome mechanical limitations while unlocking new freedoms in shape, stability, and function.

Building on this paradigm, HSHs have gained significant attention as versatile platforms for biomedical and engineering applications. They have increasingly been applied as lab‐ and organ‐on‐a‐chip systems that mimic natural tissue environments. Regulating the interconnectivity of the porous hydrogel matrix allows precise control over oxygen and nutrient permeability, as well as waste removal in 3D cultures.^[^ ^20^ ^]^ For example, hydrogels combined with commercial filters can serve as bioreactors simulating functions of the liver or lungs,^[^ ^21^ ^]^ while those bonded to metallic stents promote endothelialization and reduce restenosis.^[^ ^22^ ^]^ In the near future, with advances in the fabrication of high‐precision micro‐components using additive manufacturing, this new class of hybrid materials promises to revolutionize soft electronics,^[^ ^15^ ^]^ whilst hydrogels with spatiotemporally controlled structures offer a new generation of smart 4D materials with dynamic properties.^[^ ^19^ ^]^

However, these advanced solutions face a critical universal problem of establishing strong adhesion between the soft, hydrated hydrogels and the rigid, dry solid substrates with mismatched mechanical properties. The high water content and low surface friction of hydrogels make adhesive bonding inherently weak and unreliable.^[^ ^23^ ^]^

To address this issue, various surface modification strategies have been proposed to chemically bind hydrogels to solid materials.^[^ ^24^, ^25^, ^26^, ^27^, ^28^, ^29^, ^30^ ^]^ Examples include silane‐based surface functionalization to anchor tough hydrogel networks to metallic substrates.^[^ ^24^, ^31^ ^]^ and UV‐initiated polymerization to fabricate skin‐inspired hydrogel–elastomer hybrids.^[^ ^26^ ^]^ These methods are, however, substrate‐dependent, require pre‐treatment steps, and thus are not applicable to a wide range of material classes. The silanization approach, for example, is restricted to surfaces bearing high concentrations of hydroxyl groups. In addition, the sequence of chemical reactions involved in such wet‐chemistry methods ^[^ ^27^, ^32^ ^]^ often introduces process complexity, unwanted side‐reactions, and waste disposal issues. These methods also require extended curing processes and thus lack the immediacy essential for high‐throughput manufacturing. For instance, double‐network bonding strategies are restricted to porous solids and require up to six days to complete,^[^ ^25^ ^]^ making them incompatible with high‐throughput or environmentally conscious manufacturing. The large usage of solvents and chemicals is not environmentally sustainable and may also impede regulatory approval for biomedical applications.

In parallel, a second limitation persists: the widespread use of initiators and crosslinkers in chemically crosslinked hydrogels. While such hydrogels offer tunable mechanical strength and improved stability, the presence of residual initiators poses cytotoxic risks. Common photoinitiators such as Irgacure 2959 can degrade into reactive species that damage nucleic acids, proteins, and cell membranes, and inhibit intracellular signaling,^[^ ^33^, ^34^ ^]^ raising concerns for biomedical applications.

Dry plasma‐based techniques offer a promising alternative. We have recently demonstrated a new approach to achieve direct covalent integration of hydrogels to solid polymeric substrates using a dry, environmentally friendly plasma surface activation strategy.^[^ ^35^ ^]^ This chemical linker‐free method utilizes plasma immersion ion implantation (PIII) to bombard polymeric surfaces with energetic ions, embedding them with long‐lived reactive radicals without altering the bulk properties. The surface‐embedded radicals, stabilized in π‐conjugated nanoclusters, migrate to the surface and form covalent bonds with hydrogel molecules upon contact, enabling direct attachment of hydrogels to the surface.^[^ ^35^ ^]^ However, while eliminating the need for wet chemical processes, the PIII process is limited to carbon‐based polymeric surfaces. Given these limitations, an initiator‐free approach that enables universal, robust attachment and spontaneous crosslinking of hydrogels to virtually any solid surface, including metals and ceramics, is highly sought after.

**Figure 1:**
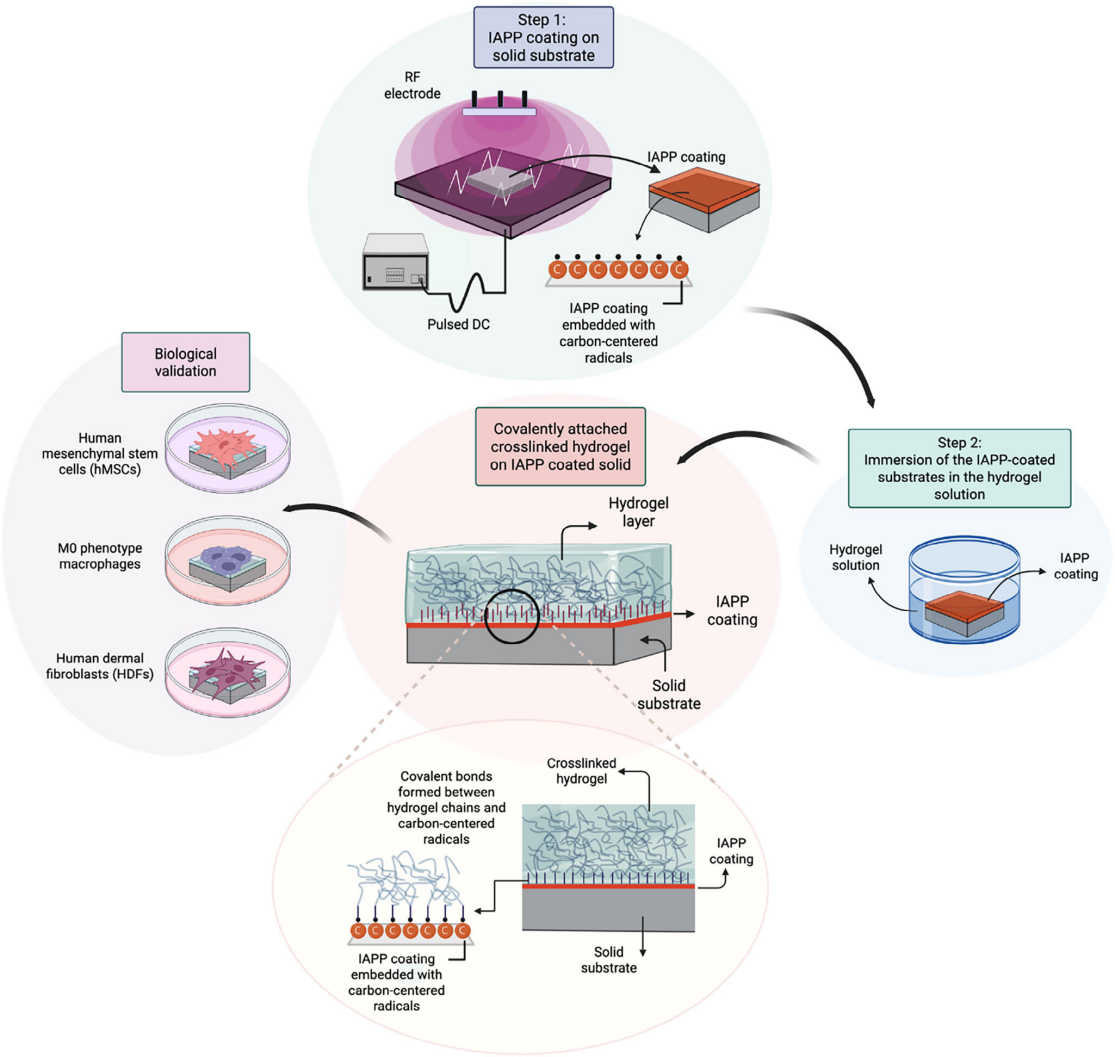
Schematic illustration of the radical‐mediated, substrate‐independent process enabled by ion‐assisted plasma polymerization (IAPP), facilitating initiator‐ and crosslinker‐free covalent attachment of hydrogels with tunable crosslinking density.

Here, we harness ion‐assisted plasma polymer (IAPP) coatings formed under high‐energy ion bombardment,^[^ ^36^, ^37^ ^]^ and introduce a substrate‐independent, initiator‐ and crosslinker‐free strategy for fabricating robust HSHs. The radicals embedded in IAPP coatings provide a versatile platform for covalent hydrogel bonding and spontaneous crosslinking, without requiring wet chemistry or chemical linkers (**Figure** **1**). This dry and scalable process enables a general approach for interface engineering, addressing long‐standing challenges in hydrogel integration. Establishing a mechanistic foundation for next‐generation biointerfaces, the platform enables tunable mechanical and biological performance, offering a transformative route toward clinically relevant materials for regenerative medicine, implantable devices, and soft tissue reconstruction.

## 2 Experimental Section

### 2.1 Materials

High‐purity acetylene (C_2_H_2_), argon (Ar), and nitrogen (N_2_) gases were procured from BOC‐Australia for ion‐assisted plasma polymerization. Titanium (Ti) sheets and stainless steel (SS) foils were sourced from Firmetal (China). High‐purity Si wafers (thickness: 515–535 µm) were purchased from TOPSIL. Low‐density polyethylene (LDPE) sheets, 0.2 mm in thickness, were obtained from Goodfellow (UK). Gelatin (porcine skin), methacrylic anhydride (MA), phosphate‐buffered saline (PBS), sodium dodecyl sulfate (SDS), chitosan (medium molecular weight), were obtained from Sigma‐Aldrich. Tyramine‐modified poly(vinyl alcohol) (PVA‐Tyr) of 2% degree of functionalization was synthesized according to previously published literature.^[^ ^38^, ^39^ ^]^ Recombinant wild‐type human Tropoelastin (TE), corresponding to residues 27–724 of GenBank entry AAC98394, was provided by Anthony Weiss, University of Sydney. Quartz slides for electron paramagnetic resonance (EPR) measurements were obtained from Helm Australia, and polystyrene tape for adhesion testing from Interpolymer Group (USA). Other materials specifically used for biological assessments will be detailed in their respective method sections.

### 2.2 IAPP Coating Deposition

**Figure 2:**
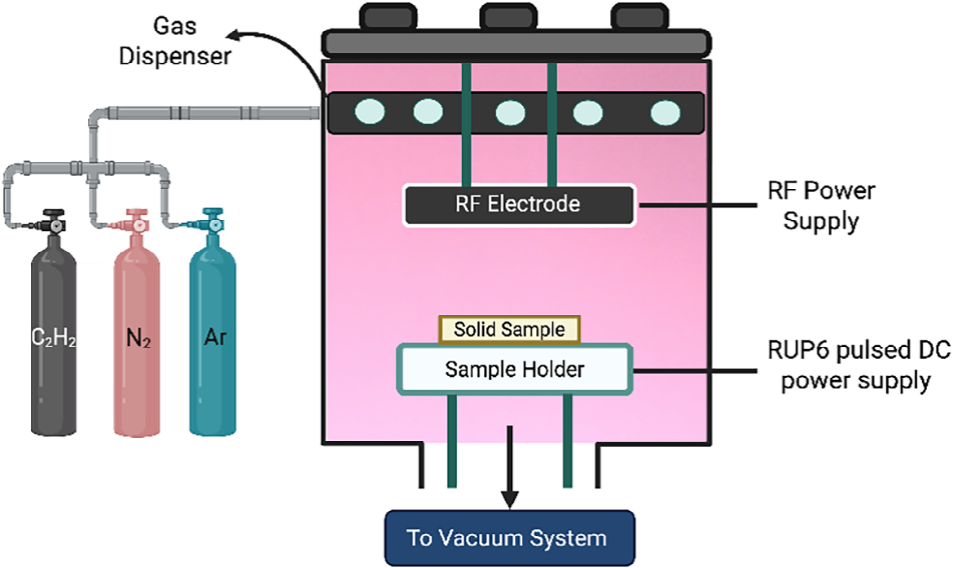
Schematic illustration of the ion‐assisted plasma polymerization system used to generate radical‐rich thin films for substrate‐independent hydrogel attachment.

Ion‐assisted plasma polymer films were deposited onto Ti, SS, glass, Si wafer, and Straumann dental implant material substrates using a custom‐designed plasma polymerization system, as previously described.^[^ ^40^, ^41^, ^42^ ^]^ Briefly, a radio frequency (RF) power supply (ENI OEM‐6AM‐1) was connected to the upper RF electrode, while a RUP6 pulsed DC power supply (GBS‐Electronik) was used to bias the substrate holder (**Figure** **2**). A reactive gas mixture of acetylene (5 sccm), argon (10 sccm), and nitrogen (15 sccm) was introduced into the chamber, maintaining a working pressure of 110 mTorr. Before deposition, base pressure was reduced to below 5 × 10^−^^2^ mTorr. Substrates were pre‐treated with argon plasma (40 sccm, RF power 50 W, substrate bias −500 V) for 10 min surface activation. Plasma polymerization was then performed using an RF power of 50 W and a pulsed DC bias of −500 V (3 kHz, 20 µs pulse duration) applied to the substrate. Deposition time was fixed at 2 min, resulting in uniform coatings with an average thickness of ≈25 nm.

### 2.3 Hydrogel–Solid Hybrid (HSH) Fabrication

Untreated and IAPP‐coated solid samples were coated with GelMA, chitosan, and PVA‐Tyr hydrogels. Rectangular samples (0.8 × 0.6 cm) were prepared and placed in 48‐well plates. GelMA (2.5% w/v) was dissolved in Milli‐Q water at 40 °C, while chitosan (2% w/v) was dissolved in 0.1m acetic acid. PVA‐Tyr was dissolved in Milli‐Q water at 65 °C at a final concentration of 2.5% w/v. Each sample was coated with 0.5 mL of hydrogel solution and incubated at room temperature for 1 h. Following incubation, samples were gently rinsed with Milli‐Q water and transferred to fresh well plates for air drying. These coated samples were subsequently used for SDS washing, stability assessments, and surface characterization via ATR‐FTIR and XPS. For ellipsometry analysis, GelMA coatings were reproduced on silicon wafer substrates.

For interfacial adhesion measurements, two sets of Ti samples (1.2 × 2.4 cm) were prepared, each comprising three replicates. In the first set, samples were submerged in a 2.5% (w/v) GelMA solution for 30 min, rinsed with Milli‐Q water, transferred to fresh well plates, and air‐dried before testing. In the second set, a 1 mL solution of GelMA (5% w/v) containing 0.5% (w/v) Irgacure was applied to the surface and photocrosslinked under UV light. Both sets were subsequently subjected to mechanical adhesion testing to assess interfacial bonding strength.

For biological assessments, untreated and IAPP‐coated Ti samples (0.8 cm × 0.6 cm) were submerged in 0.5 mL of filter‐sterilized GelMA (2.5% w/v) or PVA‐Tyr solution (2.5% w/v), with or without Tropoelastin (TE, 15 µg mL^−1^) for 1 h at room temperature. All hydrogel solutions for this set of experiments were prepared in PBS to maintain physiological osmolarity. Following incubation, samples were gently rinsed with PBS to remove excess unbound hydrogel precursors or protein. To evaluate cellular responses to GelMA crosslinked via plasma polymer layer radicals versus UV photopolymerization, IAPP‐coated Ti substrates were coated with 0.1 mL of 5% (w/v) GelMA containing 0.25% (w/v) Irgacure and exposed to UV light (wavelength: 365 nm) for 5 min to induce crosslinking.

### 2.4 Assessment of Covalent Hydrogel Attachment Via SDS Washing

To investigate the hydrogel–substrate attachment mechanism, a SDS washing protocol was employed. SDS, an anionic surfactant, selectively disrupts non‐covalent interactions while leaving covalent bonds intact.^[^ ^43^ ^]^ Hydrogel‐coated substrates—both untreated and IAPP‐modified—were incubated in 2.5% (w/v) SDS solution at 37 °C for 30 min, followed by rinsing with Milli‐Q water. Chitosan‐based HSHs were incubated in SDS solution for 1 h. Samples were then air‐dried and stored at room temperature in sealed containers before ATR‐FTIR and XPS analyses.

### 2.5 Evaluation of Long‐Term Hydrogel–Substrate Stability Under Aqueous Physiological Conditions

To examine the long‐term stability of the hydrogel–substrate attachment under physiological conditions, hydrogel‐coated samples were placed in 48‐well plates, each well containing 1 mL of PBS (pH 7.4), and incubated at 37 °C for up to 2 months. The plates were sealed with Parafilm to minimize evaporation and contamination during incubation. After 1 week, 1 month, and 2 months of incubation, samples were gently rinsed with Milli‐Q water to remove residual salts and loosely bound components. The samples were then air‐dried at room temperature and stored in sealed containers before analysis. X‐ray photoelectron spectroscopy (XPS) was conducted to assess the surface chemical composition. Both survey and high‐resolution C1s spectra were acquired to evaluate the retention of hydrogel‐specific chemical signatures and monitor potential changes in bonding states over time.

### 2.6 X‐Ray Photoelectron Spectroscopy

The surface chemistry of the HSH samples was examined before and after incubation, tape testing, and SDS washing using X‐ray photoelectron spectroscopy (XPS) (K‐Alpha+, Thermo Scientific, USA). Survey spectra were collected across −10 to 1350 eV (pass energy: 200 eV; step size: 1.0 eV; 10 scans). High‐resolution C 1s spectra were acquired over the 280–300 eV range with a 0.1 eV step size, also averaged over 10 scans. Spectral analysis and atomic quantification were conducted using Thermo Scientific Avantage software (v5.9902), with curve fitting performed via mixed Gaussian–Lorentzian functions of equal full width at half maximum (FWHM). To minimize surface contamination, untreated Ti samples were subjected to ion beam etching (IBE) at 2000 eV in monotonic mode (2.00 mm raster size) for 60 s before measurement.

### 2.7 Attenuated Total Reflectance Fourier Transform Infrared Spectroscopy (ATR‐FTIR)

Attenuated total reflectance Fourier‐transform infrared (ATR‐FTIR) spectroscopy was employed to characterize HSHs under various conditions, including before and after incubation in PBS and following SDS washing. Spectra were acquired using a Bruker Vertex 80 V spectrometer equipped with a MIRacle single‐reflection horizontal ATR accessory (PIKE Technologies, Madison, WI), featuring a germanium internal reflection element (IRE) with a 2 mm sampling area. Square samples (0.6 × 0.8 cm) were used for each measurement. Each spectrum was collected from 64 scans at a resolution of 4 cm^−1^. The IRE was cleaned thoroughly between measurements using a cotton swab moistened with isopropyl alcohol and air‐dried before recording a new background. All measurements were performed in triplicate. Spectral subtraction and data analysis were carried out using OriginPro 2022. The ATR–FTIR spectra of the underlying solid substrates (Ti, stainless steel, and glass) were subtracted from their respective IAPP‐coated counterparts to isolate the IAPP‐specific signals. Subsequently, the spectra of the IAPP coating were subtracted from those of the hydrogel‐coated samples—namely IAPP‐coated Ti+GelMA, IAPP‐coated Ti+chitosan, and IAPP‐coated Ti+PVA‐Tyr—to distinguish the spectral features of the hydrogel layers.

### 2.8 Surface Contact Angle and Surface Energy

Static contact angles were measured using the sessile drop method (Attension Theta, Biolin Scientific) with 3 µL droplets of deionized water and diiodomethane to represent polar and nonpolar liquids, respectively. For each sample, measurements were acquired at three distinct positions, and the mean values with standard deviations were reported. Side‐view images were used to determine contact angles. Surface free energy and its polar and dispersive components were calculated using the Owens–Wendt–Rabel–Kaelble (OWRK) model implemented in the Attension software.^[^ ^44^, ^45^ ^]^

### 2.9 Electron Paramagnetic Resonance (EPR) Analysis

The concentration of unpaired electrons in the IAPP coating before and after incubation in hydrogel solution was assessed using an Adani SPINSCAN X EPR spectrometer at room temperature. IAPP films were deposited on quartz samples (5 × 40 mm, 0.9 mm thickness). The spectrometer was operated using e‐Spinoza software, with the following experimental parameters: a frequency of 100 kHz, a central field of 3353 G, and a modulation amplitude of 2 G. Each sample underwent a 60 s sampling period, with 10 scans averaged to generate the final spectra.

### 2.10 Atomic Force Microscopy (AFM)

Surface morphology and root‐mean‐square (RMS) roughness of IAPP coatings on Si wafers were characterized using a PicoSPM atomic force microscope (Molecular Imaging) operating in non‐contact mode. Silicon cantilevers (PPP‐NCST‐SPL, Nanosensors) with resonance frequencies of 76–263 kHz were employed. Scans were performed with a setpoint of 2–3 V and a scan rate of 0.5 Hz using a 100 µm‐range scanner. Image processing and analysis were conducted using PicoScan 5 and WSxM software (v5.0, Nanotec Electronica).

### 2.11 Scanning Electron Microscopy (SEM)

Top‐view and cross‐sectional SEM imaging was performed using a JEOL Neoscope JCM‐6000 scanning electron microscope operated at an accelerating voltage of 10 kV in high‐vacuum mode. A secondary electron (SE) detector was used to visualize surface features and interfacial morphology. Before imaging, all samples were sputter‐coated with a thin layer of gold to enhance conductivity and minimize charging. Top‐view imaging was conducted to assess the surface morphology and coating integrity of scratched IAPP‐coated Ti substrates. For GelMA coatings, samples were prepared on glass substrates and fractured to expose cross‐sections for thickness measurements. Cross‐sectional images were acquired by SEM, and the hydrogel layer thickness was quantified using ImageJ software. The same samples were subsequently used for adhesion toughness evaluation via 90° peeling tests.

### 2.12 Interfacial Adhesion Measurements

A 90° peel test was conducted to assess the mechanical adhesion strength of thin and thick GelMA coatings on both untreated and IAPP‐coated substrates. A three‐layer structure—consisting of the substrate, GelMA hydrogel, and polystyrene tape—was assembled, with the tape applied to fully dried, hydrogel‐coated samples. Tests were performed in accordance with ISO 8510 using a universal mechanical tester (Instron 5943) at a constant peel rate of 5 mm min^−1^. Adhesion strength was defined as the maximum force per unit width of the bonded interface. To evaluate the influence of IAPP functionalization, measurements were conducted on both unmodified and coated surfaces. Following testing, samples were sealed for ATR‐FTIR analysis. Interfacial toughness (Γ_T_) was calculated using the equation:

(1) $$\Gamma_{T}=F_{\mathrm{ave}}/w$$

where F_ave_ is the average force during steady‐state peeling and w is the width of the hydrogel layer.

### 2.13 Human Mesenchymal Stromal/Stem Cell (hMSC) Isolation and Culture

hMSCs were derived from bone marrow samples collected from patients who underwent orthopedic surgery at the University Medical Centre Utrecht, Netherlands. The procedure was carried out in accordance with the approval of the institutional medical ethics committee. Human material was obtained following the principles of the Declaration of Helsinki, with approval from the local medical ethics committee at the University Medical Center Utrecht (UMCU), Utrecht, The Netherlands, under protocols METC 08–001/K and METC 07–125/C, and with written consent from the participants. The mononuclear cell fraction was isolated, and plastic‐adherent cells were expanded to passage 5. hMSCs were cultured in α‐MEM supplemented with 10% (v/v) fetal bovine serum (FBS) and 1% (v/v) penicillin/streptomycin in a humidified incubator at 37 °C with 5% CO_2_.

### 2.14 Macrophage Differentiation From Monocytes

THP‐1 human monocytic leukemia cells (American Type Culture Collection, UK) were cultured for a day in a humidified incubator (at 37 °C in 5% CO2) using RPMI 1640 culture medium (Thermo Fisher Scientific, US) supplemented with 10% (v/v) FBS and 1% (v/v) penicillin/streptomycin. Next, 100 nm phorbol 12‐myristate 13‐acetate (PMA) (Sigma‐Aldrich, Germany) was added to the cells to induce differentiation to M0 macrophages. After 2 days, the PMA‐supplemented media were removed, cells were washed with PBS, and recovered for 24 h in media without PMA.

### 2.15 Human Dermal Fibroblast (HDF) Culture

HDFs at passage 20 (American Type Culture Collection; provided by Anthony Weiss, The University of Sydney) were cultured in Dulbecco's Modified Eagle Medium (DMEM, Thermo Fisher Scientific) supplemented with 10% FBS and 100 U/mL penicillin/streptomycin. Cultures were maintained at 37 °C with 5% CO_2_ in a humidified incubator.

### 2.16 Cytotoxicity Assay

To evaluate the cytotoxicity of HSHs, the viability of hMSCs and macrophages was assessed. hMSCs (3,000 cells/cm^2^) or THP‐1‐derived M0 macrophages (50 000 cells cm^−2^) were seeded on untreated Ti, IAPP‐coated Ti, IAPP‐coated Ti+GelMA, and IAPP‐coated Ti+GelMA+Irgacure, and grown for up to 7 days. Metabolic activity was quantified as a measure of cell abundance using a WST‐8/CCK‐8 cell counting kit. Cells were assayed at defined timepoints, with results normalized to cells seeded on tissue culture plastic wells for one day. Cells were treated with 5% WST‐8 for 2 h, and absorbance was measured at 460 nm using a multimode plate reader (Clariostar, BMG Labtech).

### 2.17 Live/Dead Staining

To confirm the correlation between metabolic activity and cell viability, hMSCs and M0 macrophages were stained using a live/dead kit (Molecular Probes, ThermoScientific, US). A confocal laser scanning microscope (CSLM, Leica SP8X, Germany) was used to capture images of live (green, 500–525 nm) and dead (red, 528–640 nm) cells.

### 2.18 Evaluation of In Vitro Immune Response

THP‐1‐derived M0 phenotype macrophages were cultured using two models to evaluate potential immune reactions. In the first model, cells were directly cultured on the surface of the samples (contact‐based model) in 300 µL of RPMI 1640 culture medium (Invitrogen, USA) supplemented with 10% (v/v) fetal bovine serum (FBS) and 1% (v/v) penicillin–streptomycin (Pen‐Strep) (Invitrogen, USA). In the first model, cells were directly cultured on the surface of the samples (contact‐based model) in 300 µL of RPMI 1640 culture medium (Invitrogen, USA) supplemented with 10% (v/v) fetal bovine serum (FBS) and 1% (v/v) penicillin–streptomycin (Pen‐Strep) (Invitrogen, USA). In the second model, cells were cultured on a tissue culture plate and exposed to 300 uL conditioned medium in which samples had been incubated for a day, to assess whether an immune response was triggered by degradation products. After 1 day, cells were fixed with formalin and stained for nuclei using DAPI (Abcam, UK), and for F‐actin using Alexa Fluor 488 Phalloidin (Sigma‐Aldrich) to visualize cell morphology. Additionally, levels of the inflammatory cytokine interleukin 6 (IL‐6) were quantified in the conditioned media using a human IL‐6 ELISA kit (DuoSet, R&D Systems), following the manufacturer's protocol.

### 2.19 Cell Adhesion Assay

To assess cell adhesion on HSHs with thin hydrogel (IAPP‐coated Ti+GelMA) and thick hydrogel (IAPP‐coated Ti+GelMA+Irgacure) coatings, hMSCs were seeded onto the materials at a density of 20 000 cells cm^−^^2^. After a 24 h incubation, adherent cells were fixed with formalin and stained for F‐actin using Alexa Fluor 488 Phalloidin (Sigma‐Aldrich) and for nuclei using DAPI (Abcam, UK) for cytoskeleton visualization. Stained cells were then analyzed using CSLM.

HDFs were seeded at 4000 cells per well in a 48‐well plate on untreated Ti, IAPP‐coated Ti, IAPP‐coated Ti+GelMA, IAPP‐coated Ti+GelMA+TE, IAPP‐coated Ti+PVA‐Tyr, and IAPP‐coated Ti+PVA‐Tyr+TE. After 1, 4, and 16 h, cells were fixed with 4% (v/v) formaldehyde for 20 min at room temperature. Fixed cells were stained with ActinRed 555 ReadyProbe Reagent (Thermo Fisher Scientific) and DAPI (Thermo Fisher Scientific) for 15 min. Stained samples were mounted on glass slides using an antifade mounting medium (Thermo Fisher Scientific) and stored in the dark until imaged. Fluorescence microscopy was performed using a Zeiss AxioVert microscope equipped with filters for blue and red fluorescence. Cell adhesion was assessed by quantifying nuclei counts after 1 h of culture. Cell spreading at 1, 4, and 16 h was evaluated by measuring cell circularity using ImageJ. A total of 90 cells were analyzed per condition to assess morphological differences.

### 2.20 Cell Proliferation Assay

To evaluate cell proliferation on HSHs, HDFs were seeded at a density of 4000 cells per well in a 48‐well plate on the samples and maintained for seven days. On days 1, 4, and 7, cells were fixed with 4% (v/v) formaldehyde, stained with DAPI for 15 min, mounted with an antifade mounting medium, and stored in the dark until imaged. Fluorescence microscopy was conducted using a Zeiss AxioVert microscope with blue fluorescence filters. Images were captured from multiple sample regions and analyzed with ImageJ. Images were converted to 8‐bit grayscale, thresholded, and the watershed algorithm was used to separate overlapping nuclei. Nuclei counts from multiple fields of view were averaged per sample.

### 2.21 Statistical Analysis

Statistical analyses were performed using GraphPad Prism (v9.5.1). Two‐way ANOVA followed by Tukey's multiple comparisons test was used for cytotoxicity assessment, cell adhesion, cell proliferation, cell spreading, and for comparing XPS elemental compositions across multiple groups. One‐way ANOVA was used for comparisons involving a single XPS elemental composition, hydrogel thickness, and refractive index measurement. For interfacial toughness, statistical significance between the two groups was assessed using an unpaired, two‐tailed Student's *t*‐test. Data are presented as mean ± standard deviation from independent samples, with the number of replicates specified for each experiment. Statistical significance was denoted as follows: ns, not significant; ^*^ *p* < 0.05; ^**^ *p* < 0.01; ^***^ *p* < 0.001; ^****^ *p* < 0.0001.

## 3 Results and Discussion

### 3.1 Engineering Stable Radical‐Rich Interfaces

**Figure 3:**
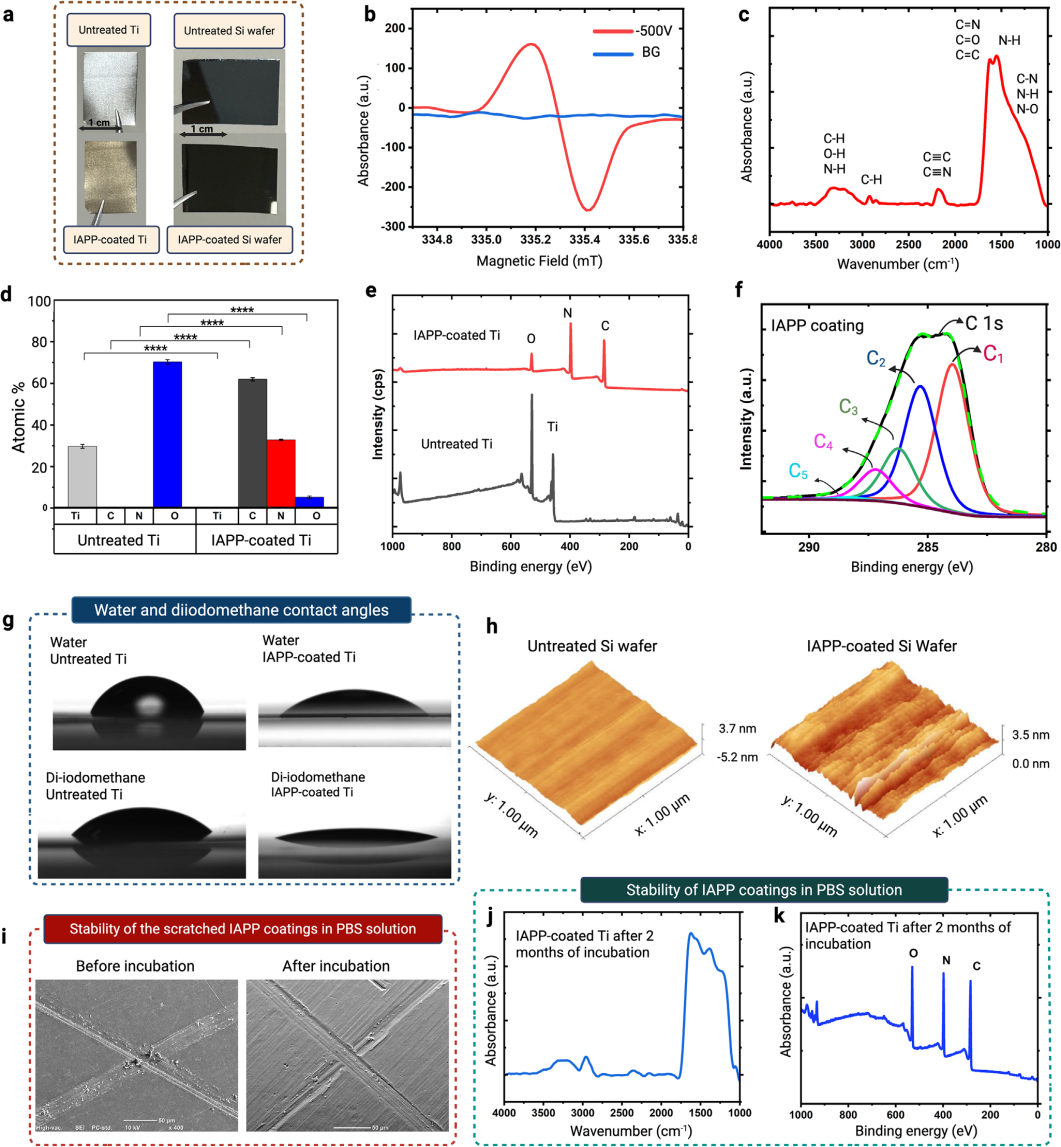
Formation and stability of radical‐rich ion‐assisted plasma polymer (IAPP) interlayers. a) Photographs of Ti and Si wafer substrates before and after IAPP coating. b) EPR spectra of untreated and IAPP‐coated quartz, showing a distinct signal indicative of embedded radicals within the IAPP layer. c) ATR‐FTIR spectra (1000–4000 cm^−1^) of IAPP‐coated Ti. d) Elemental composition from XPS survey analysis of untreated and IAPP‐coated Ti. Data are presented as mean ± SEM (*n* = 9). Statistical significance was defined as *p* < 0.05. Asterisks indicate levels of significance: ^*^ *p* < 0.05, ^**^ *p* < 0.01, ^***^ *p* < 0.001, ^****^ *p* < 0.0001.e) XPS survey spectra showing complete surface coverage of Ti after IAPP deposition. f) High‐resolution C 1s XPS spectra of IAPP coating, curve‐fitted to identify major carbon bonding environments. g) Contact angle images with water and diiodomethane on untreated and IAPP‐coated Ti.h) AFM images of uncoated and IAPP‐coated Si wafers, showing the conformal nature of the IAPP coating. i) SEM images of scratched IAPP‐coated Ti before and after two‐month incubation in PBS, showing no delamination or other forms of failure (scale bar = 50 µm). j) ATR‐FTIR spectra and k) XPS elemental composition of IAPP‐coated Ti after 2 months in PBS, confirming coating integrity.

We hypothesized that radicals embedded in a polymeric coating could be harnessed to covalently anchor and crosslink hydrogel layers onto solid surfaces regardless of their chemistry. To investigate this hypothesis, we fabricated radical‐functionalized plasma polymer films on Ti and Si wafer substrates (**Figure** **3**a), employing ion‐assisted plasma polymerization. In this process, the substrate underwent pulsed negative biasing during the plasma polymerization of a mixture of acetylene, argon, and nitrogen. The pulsed biasing strategy enhances energetic ion bombardment during plasma polymer film growth, generating radicals within the coating structure, as indicated by EPR results (Figure 3b). The EPR spectrum of the IAPP coatings shows a prominent peak at 335.2 mT (*g* = 2.0163), attributed to unpaired electrons associated with embedded radical species.

Surface chemistry and wettability are important characteristics influencing the interaction of hydrogel macromolecules with the IAP‐coated surfaces. To investigate the chemical composition of the IAPP coatings, samples were analyzed using ATR‐FTIR and XPS. Figure 3c shows ATR‐FTIR spectra obtained for the coatings deposited on Ti. The spectra confirm the formation of an organic layer exhibiting two broad absorption bands situated in the ranges of 3400–3100 cm^−1^ and 1750−1000 cm^−1^. The broad band at 3400–3100 cm^−1^ is associated with stretching vibrations of N─H, OH, and C─H,^[^ ^46^, ^47^, ^48^ ^]^ and the band at 1750−1000 cm^−1^ corresponds to C═N, C═C, C═O, and N─O, N─H, C─O, and C─N.^[^ ^47^, ^48^, ^49^, ^50^ ^]^ The peaks observed in the 3000–2800 cm^−1^ range are indicative of the asymmetric and symmetric stretching vibrations of C─H bonds.^[^ ^49^, ^50^, ^51^, ^52^ ^]^ The absorption bands at 2100─2250 cm^−1^ can be interpreted as vibration bands of the alkyne bond (C≡C) and nitrile groups (C≡N).^[^ ^46^, ^47^, ^48^ ^]^ These absorption bands are observed in all the IAPP coatings, irrespective of the substrate on which they were deposited, indicating that the coating process is substrate‐independent in terms of the obtained surface chemistry. Therefore, for further surface characterization experiments, only Ti‐coated samples were used.

The elemental composition of untreated and IAPP‐coated Ti (Figure 3d) was obtained using XPS survey spectra (Figure 3e). The IAPP coating exhibits carbon, nitrogen, and oxygen signals with atomic concentrations of 61.9% ± 0.8%, 31.8% ± 0.3%, and 6.2% ± 0.5%, respectively. XPS has a sampling depth of ≈10 nm.^[^ ^53^, ^54^ ^]^ Hence, these elemental composition data (*n* = 9), obtained from three randomly selected locations across each of three independent samples, indicate complete coverage of the Ti surface by the IAPP coating to a thickness of at least 10 nm, as evidenced by the absence of detectable Ti signals. This conclusion is consistent with our thickness measurements carried out on Si wafers using ellipsometry, which showed a coating thickness of 30 ± 2.8 nm.

Despite the absence of oxygen in the precursor gas mixture consisting of acetylene, nitrogen, and argon, the IAPP coating exhibited 6.2% ± 0.5% oxygen content. The incorporation of oxygen in the surface chemistry is attributed to an autoxidation process,^[^ ^40^, ^55^, ^56^ ^]^ which occurs when the coating is exposed to atmospheric oxygen, leading to the inevitable reaction between atmospheric oxygen and IAPP surface radicals. To gain a deeper understanding of the IAPP coating chemistry, the C 1s high‐resolution spectra were curve‐fitted (Figure 3f). The analysis identified five distinct peaks associated with C_1_ (C═C/C─H) at binding energy (BE) of approximately 284 eV, C_2_ (C─C/C─H) at BE ≅ 285.3 eV, C_3_ (C─N/C─O) at BE ≅ 286.5 eV, C_4_ (N─C═O/C═O) at BE ≅ 287.5 eV, and C_5_ (COOH) at BE ≅ 288.5 ± 0.5 eV.^[^ ^57^, ^58^, ^59^, ^60^, ^61^, ^62^ ^]^

The surface wettability of uncoated and IAPP‐coated Ti was determined by measuring static water and diiodomethane contact angles. Representative images of water and diiodomethane drops placed on the uncoated and IAPP‐coated surface are shown in Figure 3g. The IAPP coating showed a water contact angle of 40.5° ± 0.8° and surface free energy (SFE) of 54.8 ± 0.9 mN m^−1^. The mild hydrophilic character of the coating is well explained by the formation of polar nitrogen and oxygen groups as indicated by the XPS data (Figure 3c,e).

To assess the morphology of the IAPP coatings, they were deposited on atomically smooth Si wafers. AFM data (Figure 3h) provided RMS roughness values of 293.7 ± 28 and 368.6 ± 32 pm for the uncoated and IAPP‐coated Si wafers, respectively. These findings indicate that the nano‐thin layer of IAPP coating conforms to the substrate's topography.

Designing robust hybrid solid‐hydrogel structures requires a physically and chemically stable IAPP interlayer. To evaluate the coating's resistance to delamination under harsh conditions, samples were physically scratched and incubated in PBS at 37 °C for up to 2 months. SEM images taken before and after incubation (Figure 3i) showed no sign of delamination or buckling in the scratched IAPP‐coated Ti. Further supporting these observations, ATR‐FTIR spectra (Figure 3j) retained characteristic fingerprints of the IAPP coating, and XPS analysis (Figure 3k) detected no signals from the underlying Ti substrate. Such high physico‐chemical stability can be attributed to enhanced ion bombardment, particularly important during the early stages of film growth, facilitating a strong interface between the substrate and the coating through interfacial atomic mixing and the formation of carbide bonds,^[^ ^63^ ^]^ specifically in the case of carbide‐forming substrates like Ti. The stable and radical‐rich IAPP coating served as a foundation to fabricate robust hybrid‐solid‐hydrogel structures, incorporating carefully tuned degrees of crosslinking, as detailed in the following sections.

### 3.2 Initiator‐Free Hydrogel Immobilization and Crosslinking

We hypothesize that the radicals embedded in the IAPP coating serve dual purposes, generating strong covalent bonds with hydrogels and initiating spontaneous crosslinking. To validate this hypothesis, GelMA was used as a widely applied, model hydrogel. To understand the nature of bonding and attachment mechanism between GelMA and IAPP‐coated surfaces, the samples were washed with SDS detergent followed by FTIR‐ATR and XPS analyses. SDS detergent leaves covalent bonds intact while disrupting physical interactions.^[^ ^37^, ^64^, ^65^, ^66^ ^]^

**Figure 4:**
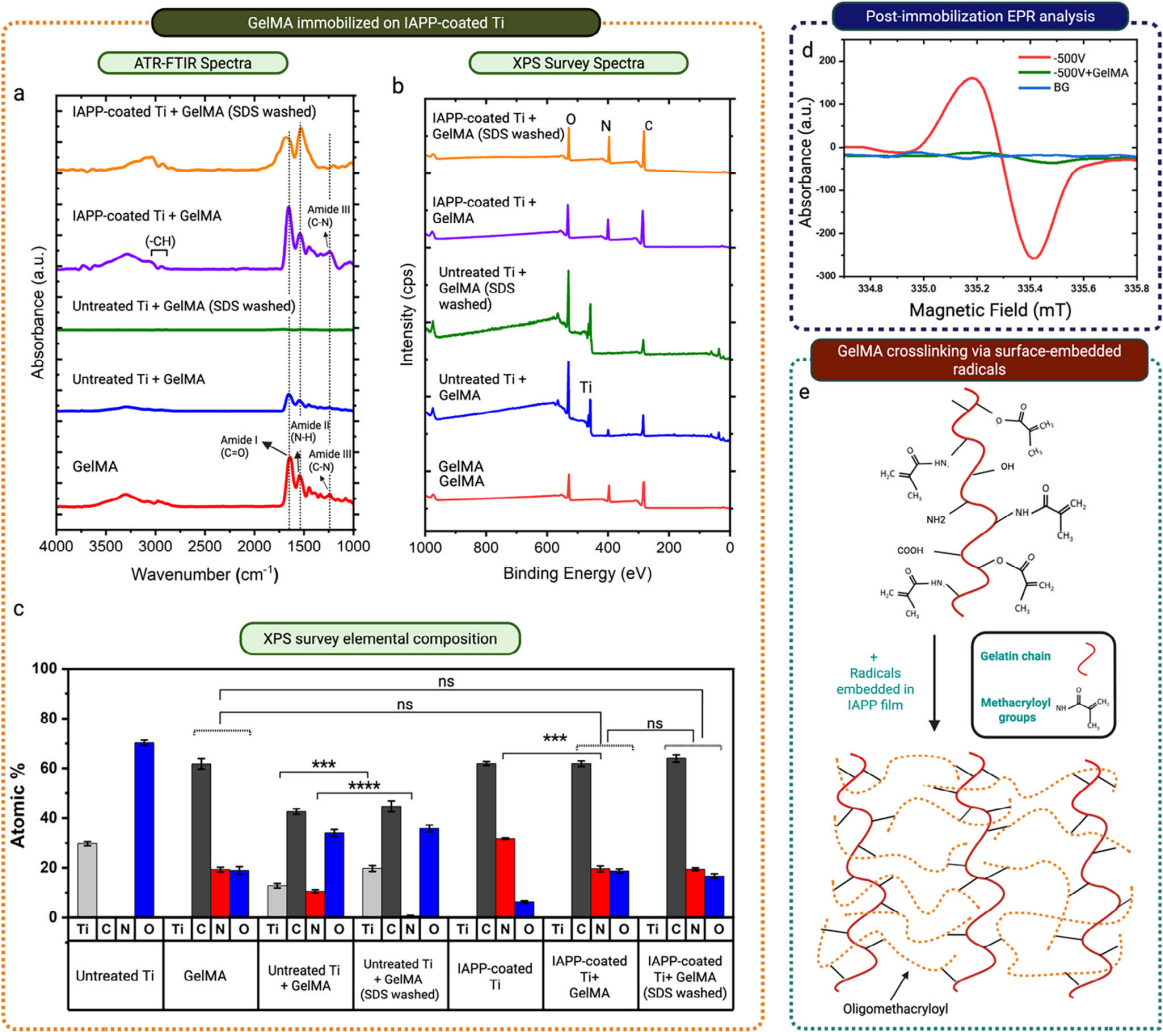
Characterization of GelMA formation, covalent attachment, and crosslinking a) ATR‐FTIR spectra (1000–4000 cm^−1^) of GelMA, IAPP‐coated Ti+GelMA, and untreated Ti+GelMA before and after SDS washing. b) XPS survey spectra, and c) corresponding elemental compositions for the same sample groups. (*n* = 6). Asterisks indicate levels of statistical significance: ^*^ *p* < 0.05, ^**^ *p* < 0.01, ^***^ *p* < 0.001, ^****^ *p* < 0.0001. d) EPR spectra of IAPP‐coated films before and after hydrogel incubation; BG represents the background signal from untreated quartz. e) Schematic of radical‐initiated crosslinking mechanism of GelMA enabled by the long‐lived radicals embedded in the IAPP coating.

The FTIR‐ATR spectra of samples before and after SDS washing are shown in **Figure** **4**a, alongside the ATR‐FTIR spectrum of GelMA as a control. The control spectrum was acquired from a 1 ml application of a 2.5% w/v GelMA solution deposited on a glass substrate and measured before any washing procedure. The broad band at 3330–3100 cm^−1^ is attributed to OH (3200–3350 cm^−1^), N─H (≈3344 cm^−1^ and ≈3202 cm^−1^), and C─H (≈3080 cm^−1^ and ≈2940 cm^−1^),^[^ ^46^, ^47^, ^48^ ^]^ while the band at 1750–1000 cm^−1^ is associated with C═O stretching (≈1650 cm^−1^ amide I), C═C (≈1620 cm^−1^), N‐H bending (≈1538 cm^−1^, amide II), C─H deformation vibration (≈1450 cm^−1^), and C─N stretching (≈1220 cm^−1^, amide III).^[^ ^35^, ^46^, ^67^, ^68^, ^69^, ^70^ ^]^ The ATR‐FTIR spectra of the GelMA synthesized in this study exhibited two broad absorption bands (red spectrum in Figure 4a) in the ranges of 3330–3100 cm^−1^ and 1750–1000 cm^−1^, which are in agreement with the characteristic spectral features of GelMA reported in the literature.^[^ ^69^, ^71^ ^]^

Upon incubation of uncoated Ti samples in GelMA solution, low‐intensity characteristic peaks were initially detected (blue spectra, Figure 4a), but they were removed following SDS washing. In contrast, IAPP‐coated Ti samples exhibited strong, well‐defined peaks after GelMA incubation, which persisted even after SDS treatment (purple and orange spectra, Figure 4a). SDS washing slightly shifted the position and altered the shape of the amide absorption peak, indicating unfolding or conformational changes of the hydrogel molecules,^[^ ^35^, ^72^ ^]^ without their removal from the surface. This observation, coupled with the higher intensity of the GelMA‐related peaks on IAPP‐coated Ti+GelMA in comparison to untreated Ti+GelMA and their persistence after SDS washing, suggests the formation of a crosslinked GelMA hydrogel layer with covalent interactions enabled by the IAPP coating.

The covalent nature of bonding between the GelMA hydrogel and the IAPP coating is reinforced by the XPS survey spectra (Figure 4b) and calculated elemental compositions (Figure 4c). The elemental composition of IAPP‐coated Ti+GelMA shows a nitrogen atomic concentration of 19.6 ± 1.1 atm%, closely resembling that of GelMA (19.3 ± 0.9 atm%), which remains almost unchanged after SDS washing (19.4% ± 0.6 atm%). In contrast, the initial nitrogen content of 10.5 ± 0.7 atm% measured for untreated Ti in the GelMA solution was reduced to almost 0 atm% (0.6% ± 0.2 atm%) after SDS washing, indicating the complete detachment of physically adsorbed GelMA from the untreated Ti substrate. The detection of 12.8 ± 0.9 atm% Ti on untreated Ti+GelMA suggests that the thickness of the GelMA layer is less than 10 nm, which is the sensitivity depth of XPS.^[^ ^54^, ^73^, ^74^ ^]^ In contrast, the resemblance in the elemental composition of IAPP‐coated Ti+GelMA after SDS washing to that of GelMA indicates that GelMA forms a layer that is thicker than 10 nm. This conclusion aligns well with our GelMA thickness measurements using ellipsometry conducted on untreated silicon wafer + GelMA and IAPP‐coated silicon wafer + GelMA before washing with SDS detergent. Measured GelMA thickness was 9 ± 1.1 nm on untreated wafers and 26.8 ± 1.9 nm on IAPP‐coated wafers. Taken together, these results provide strong evidence that the interaction between GelMA and the IAPP coating is covalent, contrasting with the physical interaction observed on uncoated substrates.

The covalent interaction between GelMA hydrogels and IAPP coating is explained by the reactive radicals embedded in the plasma polymerized coating, as informed by the EPR data (Figure 3b). To verify this mechanism, we measured the EPR signals after the hydrogel was formed on the surface (Figure 4d). Significant quenching of radicals was observed compared to the EPR signals from the IAPP coating alone, indicating their termination through reactions with the GelMA hydrogel. This reduction in intensity supports covalent reactions between the radicals and GelMA.

Beyond facilitating covalent bonding at the interface, the radicals embedded in the IAPP coating also initiate the crosslinking of GelMA hydrogels, removing the need for external initiators or crosslinkers – agents often associated with cytotoxicity when incompletely reacted.^[^ ^75^ ^]^ The radical‐initiated crosslinking mechanism initiated from the solid state can proceed by cleaving the π component of the C═C bond, converting it into a single (C─C) or sigma bond.^[^ ^76^ ^]^ Figure 4e illustrates this proposed mechanism, where radicals emerging from the surface induce crosslinking by breaking the C═C bonds in methacryloyl groups, leading to covalent linkages between gelatin chains.^[^ ^69^, ^77^, ^78^ ^]^ Detection of changes in the C═C stretching region of GelMA by ATR‐FTIR spectra is challenging due to its close proximity (≈1620 cm^−1^) to the strong C═O stretching band of amide I (≈1650 cm^−1^). However, cross‐link formation can be inferred from changes in the intensity ratio of the C─H stretching band (3100–3000 cm^−1^) to the C═O band (≈1620 cm^−1^) in the ATR‐FTIR spectra.^[^ ^69^ ^]^ For IAPP‐coated Ti+GelMA, the C─H/C═O peak ratio was 0.851 ± 0.046, substantially higher than the 0.171 ± 0.032 measured for uncrosslinked GelMA, indicative of the formation of cross‐links. Such changes in the chemical structure of GelMA are in good agreement with a recent study, which reported a lower concentration of C─H bonds in uncrosslinked GelMA compared to samples crosslinked using Irgacure 2959 and UV exposure for 1 min.^[^ ^69^ ^]^ Taken together, it can be concluded that radical‐rich IAPP coatings facilitate the covalent attachment of GelMA hydrogel to Ti surfaces and act as a solid reservoir for radical‐mediated crosslinking within the hydrogel matrix.

### 3.3 Aqueous Stability of Covalently Anchored Hydrogels

Hydrogel layers must demonstrate long‐term stability in aqueous environments to be viable for almost any application in tissue regeneration and as implant coatings. To assess the stability and robustness of the GelMA coatings, the samples were incubated in a PBS solution at 37 °C for up to 2 months followed by XPS analysis.

**Figure 5:**
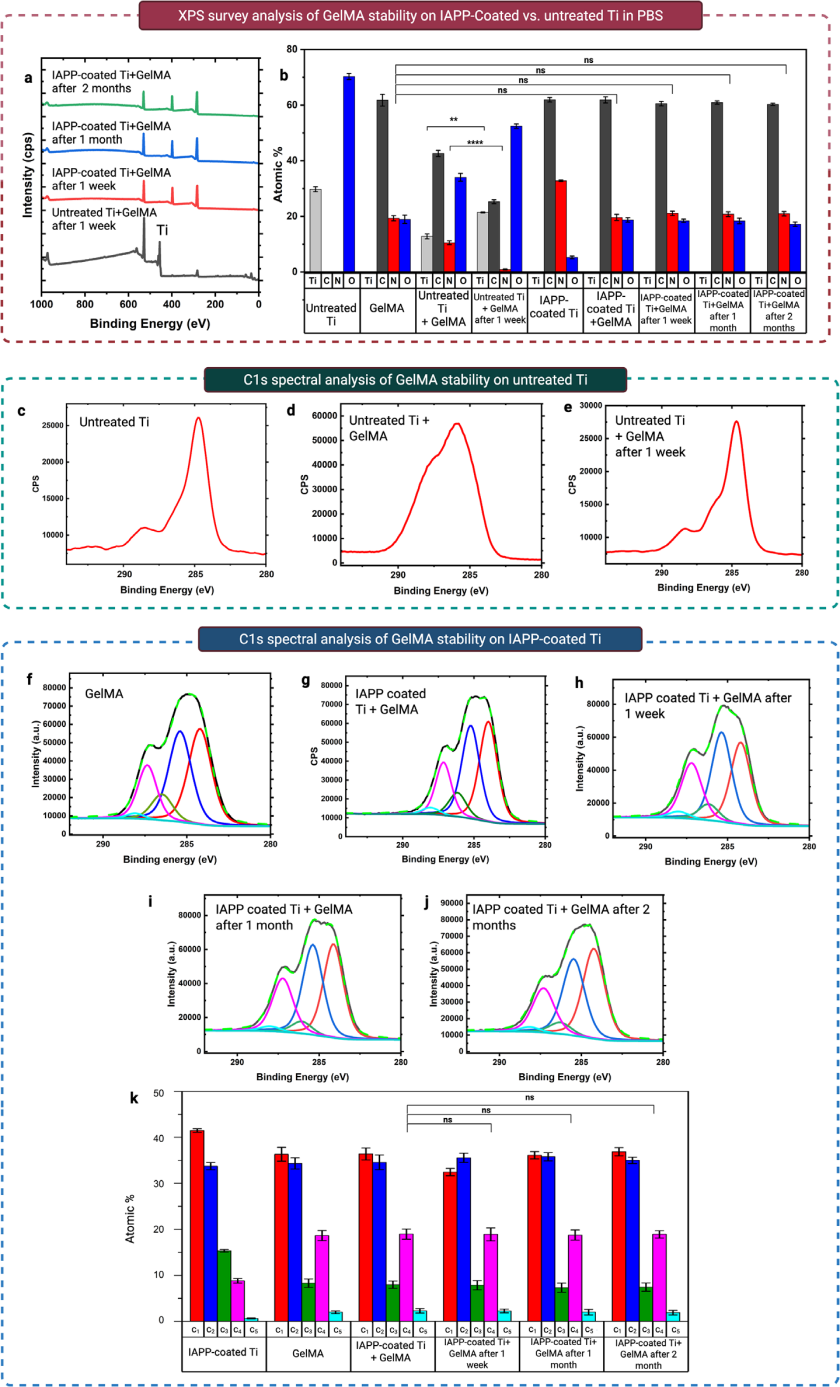
Long‐term aqueous stability of covalently anchored GelMA. a) XPS survey spectra of untreated Ti+GelMA after 1 week and IAPP‐coated Ti+GelMA after 1 week, 1 month, and 2 months of PBS incubation. b) Atomic composition of Ti, GelMA, untreated Ti+GelMA before and after 1 week, and IAPP‐coated Ti+GelMA before and after 1 week, 1 month, and 2 months. c–e) C 1s high‐resolution spectra of untreated Ti, untreated Ti+GelMA, and untreated Ti+GelMA after 1 week. f–j) C 1s spectra of GelMA, IAPP‐coated Ti+GelMA before and after 1 week, 1 month, and 2 months. k) Relative area percentages of peak fitted C 1s components: C_1_ (C─C(sp^3^/C─H) at 284 eV, C_2_ (C─C(sp^2^/C‐H) at 285.5 eV, C_3_ (C─N/C─O) at 286.5 eV, C_4_ (N─C═O/C═O) at 287.5 eV and C_5_ (COOH) at 288.5 eV. Data are expressed as mean ± SEM (*n* =  6). Statistical significance is denoted by asterisks: ^*^ *p* < 0.05, ^**^ *p* < 0.01, ^***^ *p* < 0.001, ^****^ *p* < 0.0001.

The XPS survey spectra and the calculated elemental composition for the samples before and after incubation are shown in **Figure** **5**a,b, respectively. After one week of incubation, the nitrogen content on untreated Ti+GelMA dropped from 10.5 ± 0.7 atm% to 0.9 ± 0.2 atm%, while the Ti signal increased from 12.8 ± 0.9 atm% to 19.9 ± 1.2 atm%, indicating near‐complete removal of the physically adsorbed GelMA layer. In contrast, no Ti signal was detected for IAPP‐coated Ti+GelMA, and the nitrogen content remained stable at ≈20% after incubation. Carbon and oxygen levels also showed minimal change, closely matching those of GelMA alone, indicating strong, long‐term stability of the hydrogel layer on the IAPP‐coated surface.

The long‐term stability of the hydrogel coatings is further supported by the C1s high‐resolution XPS spectra (Figure 5c–j). For untreated Ti+GelMA, notable spectral changes after one week in PBS (Figure 5e) resemble those of bare Ti (Figure 5c), indicating GelMA detachment. In contrast, the IAPP‐coated Ti+GelMA samples retained their spectral features throughout the 2‐month incubation. Curve‐fitting of the C1s spectra identified five components: C_1_ (C═C/C–H, ≈284 eV), C_2_ (C─C/C─H, ≈285.3 eV), C_3_ (C─N/C─O, ≈286.5 eV), C_4_ (N─C═O/C═O, ≈287.5 eV), and C_5_ (COOH, ≈288.5 ± 0.5 eV).^[^ ^57^, ^58^, ^59^, ^60^, ^61^, ^62^ ^]^ The area percentage of these components, plotted in Figure 5k, shows no significant differences for the IAPP‐coated Ti+GelMA samples before and after incubation in PBS for the durations of 1 week to 2 months. Such high aqueous stability is attributed to covalent bonding between GelMA and the radical‐rich IAPP interface, as discussed in Section 3.2.

### 3.4 Radical‐Rich Interfaces Enable Tunable Hydrogel Crosslinking and Thickness Without Extra Reagents

We demonstrated that radicals embedded in the IAPP coating enable reagent‐free cross‐linking and covalent attachment of hydrogels to Ti with long‐term stability. The concentration of radicals within the IAPP film structure can be modulated by tuning the degree of ion bombardment. This is achieved by simply tuning the pulsed negative bias voltage applied to the substrate during the deposition of the plasma polymer layer, as previously explained in detail.^[^ ^42^ ^]^

**Figure 6:**
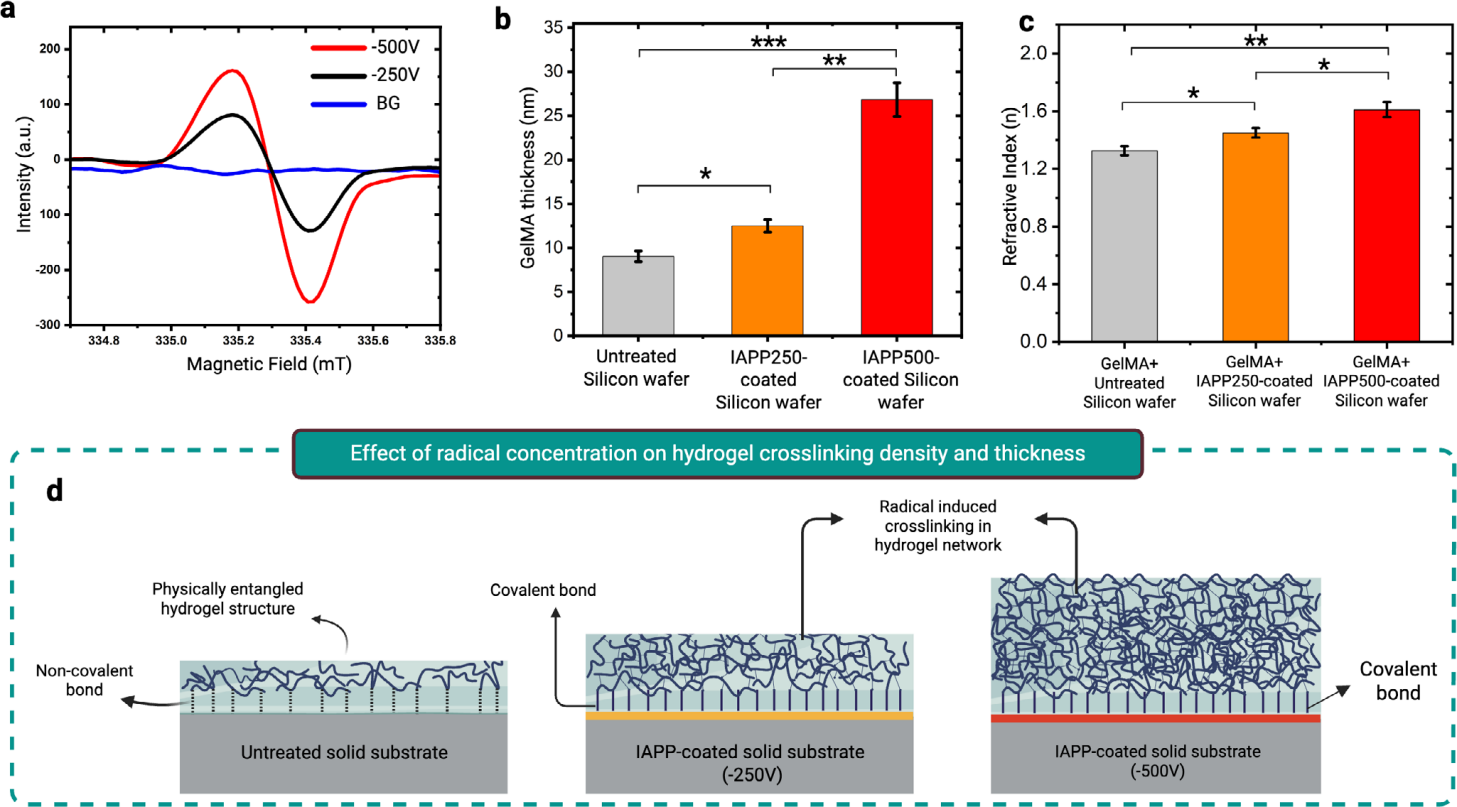
Radical‐rich interfaces enable tunable hydrogel crosslinking and thickness without extra reagents. a) EPR spectra of IAPP films deposited at biasing voltages V_b_ = −250 and −500, indicating the presence of a higher concentration of radicals in the coating deposited at the higher bias voltage. b) Comparison of the thickness of GelMA layers formed on untreated silicon wafers and IAPP‐coated silicon wafers deposited at V_b _= −250 and −500. c) Comparison of the refractive index (n) of GelMA hydrogel layers formed on untreated silicon wafers and IAPP‐coated silicon wafers deposited at V_b_ = −250 and −500. Data are presented as mean ± SEM (n  =  3). Asterisks indicate statistical significance as follows: ^*^p < 0.05, ^**^p < 0.01, ^***^p < 0.001, ^****^p < 0.0001. d) Schematic illustration of the relationship between radical concentration in IAPP coatings and the resulting crosslinking density and thickness of the hydrogel layer.

The EPR spectra of the IAPP films polymerized at bias voltages of −250 and −500 V are presented in **Figure** **6**a. The deposition times applied for the preparation of these coatings were accordingly adjusted to achieve a constant thickness of 30.0 ± 3.0 nm. Double integration of EPR spectra and comparison with an untreated sample indicates that the coating deposited under a pulsed bias of −500 V exhibited a 40.7% higher concentration of unpaired electrons compared to the coating deposited under a −250 V bias. The higher concentration of unpaired electrons (i.e. radicals) arises from more extensive hydrocarbon fragmentation, bond cleavage, and chain scission of the growing plasma polymer, induced by intensified ion bombardment at elevated bias voltages.^[^ ^42^ ^]^

To investigate the functional impact of differing radical concentrations, and to elucidate how varying fluxes of radicals emerging from the IAPP surfaces influence hydrogel thickness and crosslinking degree, Si wafer samples were coated with IAPP at two bias voltages (−250 and −500 V). The samples were then immersed in a 2.5% GelMA solution at 37 °C without additional reagents. Following rinsing, ellipsometry was employed to assess both hydrogel thickness and crosslinking degree by measuring the refractive index (n).

The thicknesses of the resulting thin hydrogel layers formed on the uncoated and IAPP‐coated surfaces are shown in Figure 6b. GelMA formed a significantly thicker layer on surfaces prepared with a bias voltage of −500 V, measuring 25.8 ± 1.9 nm, compared to the thinner layer of 12.5 ± 0.9 nm observed on surfaces prepared with a bias voltage of −250 V.

The degree of crosslinking/density of thin films can be informed by the changes in refractive index (n).^[^ ^63^, ^79^, ^80^ ^]^ Higher crosslinking increases hydrogel densification, resulting in elevated n values.^[^ ^63^, ^79^, ^80^ ^]^ GelMA formed on the IAPP coating prepared with a bias voltage of −500 V exhibited a significantly higher refractive index (*n* = 1.61 ± 0.05) compared to that on the IAPP coating prepared with a bias voltage of −250 V (*n* = 1.45 ± 0.05) (Figure 6c). The refractive index of GelMA on the untreated surface was the lowest, at *n* = 1.32 ± 0.04.

The increase in hydrogel thickness and refractive index for higher concentrations of surface‐embedded radicals may arise from two complementary effects. First, the radicals migrate from the bulk to the surface,^[^ ^36^, ^56^ ^]^ forming covalent bonds with hydrogel chains at the interface. A higher flux of radicals emerging to the surface thus results in a greater density of surface‐attached hydrogel chains,^[^ ^56^ ^]^ which in turn leads to enhanced interaction and entanglement, forming denser hydrogel layers with a larger refractive index as schematically illustrated in Figure 6d. Second, the radicals can diffuse into the hydrogel solution, similar to their behavior in aqueous ^[^ ^81^ ^]^ and other liquid environments such as ionic liquids, glass‐forming solvents, and hydrogen‐bonding liquids,^[^ ^82^ ^]^ where they react with methacrylate groups on GelMA to further drive crosslinking. Such a radical‐driven mechanism, initiated from the solid state, enables tuning of both hydrogel thickness and crosslinking degree by simply adjusting the biasing voltage during plasma polymer film growth, without the need for any external reagents.

The ability to tune hydrogel thickness and crosslinking density through the concentration of surface‐embedded radicals offers a promising platform for controlled release from a surface‐bound hydrogel. Such robust coatings can serve as matrices where sensitive therapeutics are loaded, while variations in crosslinking density and layer thickness provide a means to modulate release profiles. The nanometre‐scale hydrogel layers are advantageous where preserving the underlying surface morphology is required. They can present biochemical and biomechanical cues for optimal cellular responses without clogging pores or disrupting device nano/micro architecture.^[^ ^83^ ^]^ This is particularly of interest for nanostructured systems, including nanotubular titanium, where device performance depends on retaining the native surface nanotopography, and porous meshes, where maintaining open pores is needed for functionality.^[^ ^84^, ^85^ ^]^

Where thicker hydrogel coatings are required, the covalently anchored nano layer formed on the IAPP coating can act as a mechanically stable primer for further hydrogel growth. Alternatively, micrometre to millimetre scale hydrogel layers can be formed directly on IAPP‐coated substrates by incorporating photoinitiators into the precursor solution, as demonstrated in the next section.

### 3.5 Peel and Lap Shear Tests Reveal Robust Hydrogel–Solid Adhesion in Both Hydrated and Dehydrated States

**Figure 7:**
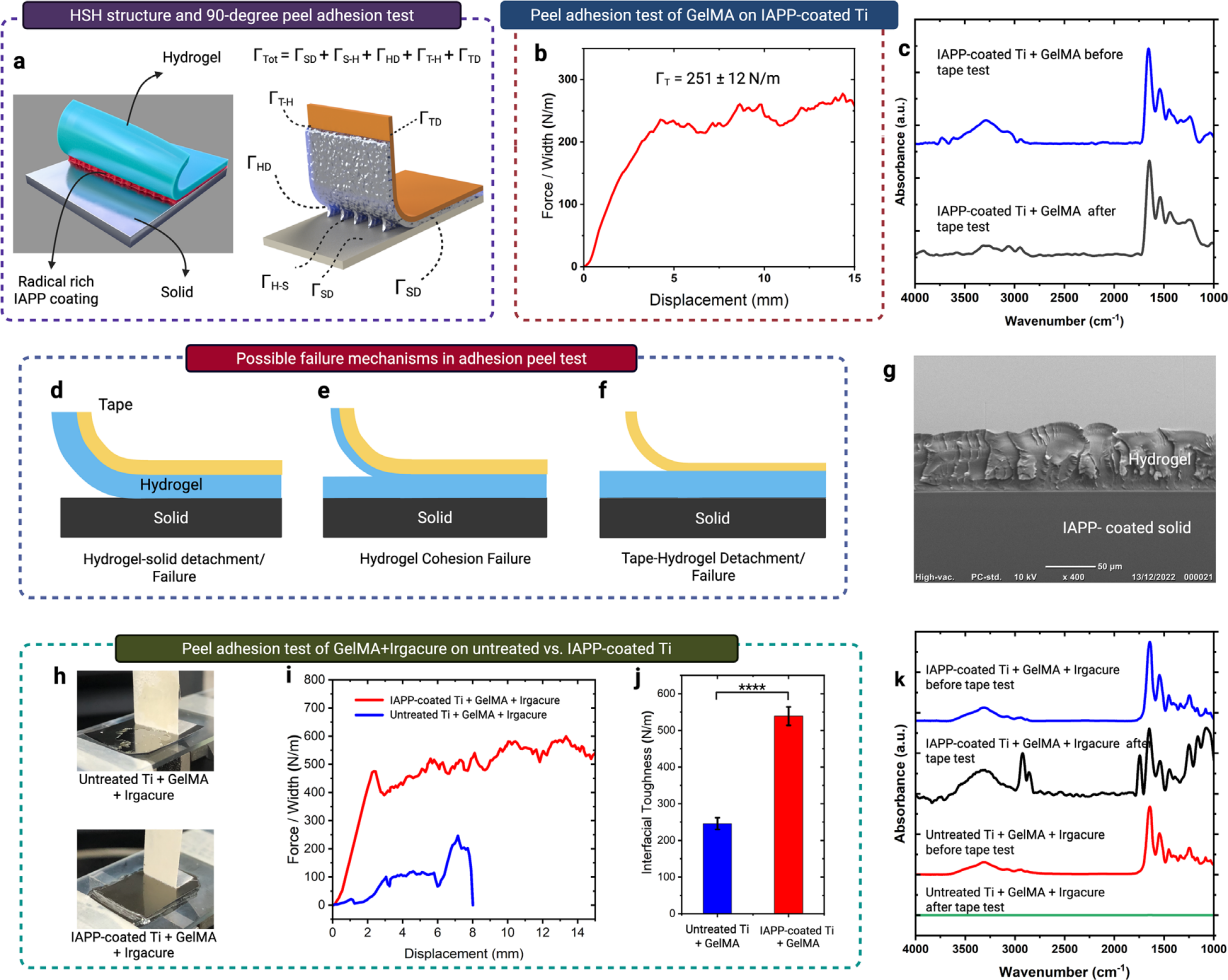
Interfacial adhesion assessment of HSHs. a) Schematic illustration of the HSHs and 90‐degree peel adhesion test. b) Peeling force per width of tape versus displacement curves and corresponding interfacial toughness (Г_Tot_) value for IAPP‐coated Ti+GelMA. c) ATR‐FTIR spectra of IAPP‐coated Ti+GelMA before and after tape testing. d–f) Schematic representations of possible failure scenarios during the peel test. g) SEM cross‐sectional image showing the thickness of UV‐crosslinked GelMA (with Irgacure) on IAPP‐coated glass. h) Photographic images captured during the peel test for thick hydrogel coatings. i) Peeling force–displacement curves and j) corresponding Г_Tot_ values for UV‐crosslinked GelMA on untreated and IAPP‐coated Ti. Data are shown as mean ± SEM (*n* = 9). Statistical significance is indicated by asterisks: ^*^ *p* < 0.05, ^**^ *p* < 0.01, ^***^ *p* < 0.001, ^****^ *p* < 0.0001. k) ATR‐FTIR spectra of surfaces post‐testing, highlighting GelMA retention on IAPP‐coated Ti and detachment from untreated Ti.

Strong adhesion of hydrogels to solid surfaces is required for the functionality of implantable medical devices, particularly in weight‐bearing applications such as orthopedic and dental implants.^[^ ^43^ ^]^ To quantify the adhesion strength of GelMA hydrogels on IAPP‐coated Ti surface, 90‐degree peel tests were performed using standard polystyrene tape strips. The tape was applied to hydrogel‐coated samples, followed by controlled peeling to measure the total interfacial toughness (Г_Tot_), as illustrated in **Figure** **7**a. ATR‐FTIR analysis was then used to determine the failure mode and the origin of adhesion loss.

During peeling, the applied force is dissipated through five distinct pathways: i) mechanical deformation of the substrate itself (Г_SD_), ii) rupture of interfacial bonds between the hydrogel and the solid substrate (Г_H‐S_), iii) internal energy dissipation within the hydrogel bulk due to the breakage of chemical bonds and plastic energy dissipation within hydrogel network (Г_HD_), iv) rupture of interfacial bonds between the tape and the hydrogel (Г_T‐H_), and (v) mechanical deformation of the tape itself (Г_TD_). Thus, the total interfacial toughness is the sum of these components ^[^ ^24^ ^]^:

(2) $$\Gamma_{\mathrm{Tot}}=\Gamma_{\mathrm{SD}}+\Gamma_{S-H}+\Gamma_{\mathrm{HD}}+\Gamma_{T-H}+\Gamma_{\mathrm{TD}}$$

Given the negligible deformability of Ti (Г_SD_ ≈ 0) and tape (Г_TD_≈ 0), the measured toughness could reflect interfacial adhesion between hydrogel and solid, hydrogel bulk dissipation, and interfacial adhesion between hydrogel and tape (Г_Tot_ ≈ Г_H‐S_ + Г_HD_+ Г_T‐H_). The peeling force per unit width versus displacement curve, and the corresponding interfacial toughness (Г_Tot_), for the IAPP‐coated Ti+GelMA sample are shown in Figure 7b, with a measured Г_Tot_ of 251 ± 12 N m^−1^. ATR‐FTIR spectra before and after the tape test (Figure 7c) show that GelMA peaks remained present on the IAPP‐coated samples following peeling.

The persistence of GelMA ATR‐FTIR peaks after the tape test on IAPP‐coated samples confirms that the hydrogel remained stably anchored, and failure occurred either within the hydrogel network (cohesive failure) or at the tape–hydrogel interface (Figure 7e,f), rather than at the hydrogel–solid interface (Figure 7d). As such, the measured toughness for IAPP‐coated samples does not reflect the true interfacial adhesion energy but rather provides a lower bound, implying that the actual interfacial toughness (Γ_H‐S_) is higher than the measured value.

We demonstrated that initiator‐free GelMA hydrogels with a thickness of 25.8 ± 1.9 nm can be formed on IAPP coatings fabricated under the greatest energy ion bombardment conditions (Figure 6b). While such thin hydrogel coatings in the nanometer scale offer significant advantages in terms of interfacial integrity, responsiveness, and fabrication precision, certain biomedical applications, such as blood‐contacting devices,^[^ ^86^ ^]^ may benefit from thicker hydrogel layers in the micro‐ to millimeter range to meet specific functional requirements. To assess whether IAPP coatings can also support robust adhesion of such thick hydrogels, we prepared UV‐crosslinked GelMA coatings by incorporating Irgacure (0.1% w/v) into a 5% w/v GelMA solution, producing coatings with a thickness of 57 ± 15 µm (Figure 7g). The HSHs incorporating such micrometer‐thick hydrogel coatings, on both untreated and IAPP‐coated Ti surfaces, were subjected to 90‐degree peel adhesion tests. Photographic images during testing are shown in Figure 7h. The corresponding peeling force–displacement curves and measured interfacial toughness values (Г_Tot_) are presented in Figure 7i,j, while ATR‐FTIR spectra of the surfaces before and after peeling are shown in Figure 7k. The UV‐crosslinked GelMA hydrogel, physically attached to Ti, could be removed readily from untreated Ti (Figure 7h), whereas it remained strongly adhered and intact on IAPP‐coated Ti. The measured Г_Tot_ was 539 ± 25 N m^−1^ for IAPP‐coated samples and 246 ± 16 N m^−1^ for untreated Ti.

The measured Г_Tot_ of 246 ± 16 N m^−1^ on untreated Ti indicates that GelMA can develop detectable physical adhesion. This is due to the protein‐derived chemistry of GelMA and the intrinsic characteristics of Ti, where the hydroxyl‐rich oxide surface layer allows for physical adsorption of protein‐based macromolecules via hydrogen bonding, van der Waals, and/or polar interactions, aided by nanoscale surface features that can result in physical interlocking.^[^ ^87^ ^]^ However, these interactions are inherently weak, manifested as a markedly lower interfacial strength than that achieved with IAPP modification, where Г_Tot_ increases to 539 ± 25 N m^−1^, over twice the adhesion measured on bare Ti.

ATR‐FTIR confirmed the loss of GelMA peaks from untreated Ti (green spectra in Figure 7k, corresponding to the detached regions in Figure 7h) and their persistence across the IAPP‐coated surface (black spectra). The appearance of additional peaks (≈1750 cm^−1^ and within the 1300–1000 cm^−1^ region in the ATR‐FTIR spectra) could be due to tape residue left on the GelMA after peeling. The disappearance of GelMA peaks from the Untreated Ti+GelMA+Irgacure sample indicates that the failure occurred at hydrogel and solid interface (Figure 7d). In contrast, the presence of GelMA peaks after the adhesion test on IAPP‐coated Ti+GelMA+Irgacure samples suggests that failure occurred either within the hydrogel network (cohesive failure; Figure 7e) or at the hydrogel–tape interface (Figure 7f). However, the appearance of additional peaks corresponding to tape residues suggests interfacial failure at the hydrogel–tape boundary (Figure 7f).

Since failure occurred either within the hydrogel network (Г_HD_) or at the tape–hydrogel interface (Г_T‐H_), the true interfacial bonding strength (Г_Tot_) between GelMA and the IAPP‐treated surface is expected to exceed the measured value of 539 ± 25 J m^−^^2^. The peel adhesion results, together with the persistent GelMA signals post‐testing, correlate well with the long‐term aqueous stability performance (Section 3.3) and are underpinned by the radical‐induced covalent bonding discussed in Section 3.2.

To further investigate the robustness of GelMA coatings, we conducted lap shear tests on dried GelMA hydrogel formed between two IAPP‐coated LDPE substrates prepared following the procedure detailed in the Supporting Information. LDPE was selected for this experiment because its optical transparency allows UV‐light photopolymerization of the GelMA sandwiched between the two substrates, which is not possible with opaque metal surfaces such as Ti. During the sample preparation, the dried hydrogel detached completely from the untreated LDPE surface, making it impossible to test the materials and obtain adhesion strength. In contrast, when the same test was performed using IAPP‐coated LDPE substrates, the dried GelMA firmly attached to the samples. We note that LDPE is highly stretchable and absorbs part of the applied energy. Therefore, the measured value (≈110 kPa, Figure S1 a,b, Supporting Information) reflects a combination of substrate deformation and interfacial adhesion.

As hydrated stability is also important for applications such as diagnostic tools, cell‐culturing platforms,^[^ ^88^ ^]^ and implantable hydrogel coatings ^[^ ^89^ ^]^ we assessed adhesion under physiologically relevant conditions. GelMA–LDPE sandwich samples were rehydrated in Milli‐Q water for 4 h before testing. Lap‐shear measurements showed that hydrated GelMA anchored to IAPP‐treated LDPE, through an evaporation‐induced enhanced concentration mechanism,^[^ ^90^ ^]^ demonstrating a strong adhesion strength with a maximum shear stress of 61 kPa (Figure S1a,b, Supporting Information). In contrast, samples prepared on untreated LDPE showed negligible adhesion, with the hydrogel detaching immediately upon loading. These findings demonstrate robust covalent anchoring of hydrogels to IAPP‐coated Ti substrates, significantly enhancing interfacial integrity under mechanical stress.

### 3.6 Universal Applicability Across Diverse Hydrogels and Solid Surfaces

**Figure 8:**
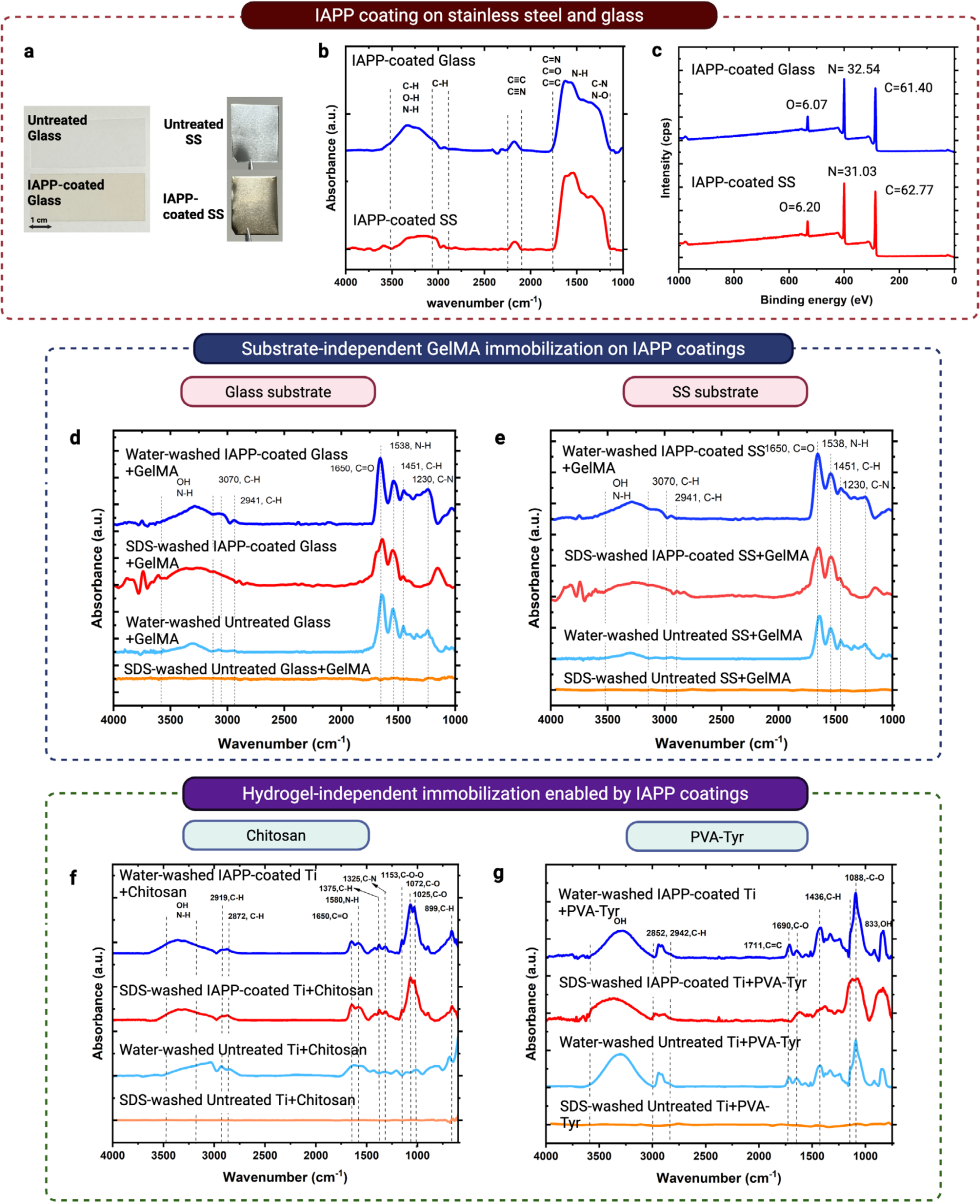
Substrate‐ and hydrogel‐independent fabrication of HSHs enabled by IAPP. a) Representative photographs of glass and stainless steel (SS) substrates before and after IAPP coating. b) ATR‐FTIR spectra of IAPP‐coated glass and SS. c) XPS survey spectra of IAPP‐coated glass and SS. d,e) ATR‐FTIR spectra of GelMA immobilized on IAPP‐coated and untreated glass and d) SS, e) before and after SDS washing. f,g) ATR‐FTIR spectra of chitosan f) and PVA–Tyr g) attached to IAPP‐coated and untreated Ti, before and after SDS washing.

For broad adoption, any strategy applied to fabricate HSH materials must be applicable across a wide range of hydrogel chemistries and solid substrate types. To demonstrate that GelMA‐Ti was not an isolated case and to establish the capability of the IAPP approach in enabling substrate‐independent hydrogel integration, we also fabricated HSHs on stainless steel (SS) and glass, two solid materials commonly used in biomedical and electronic applications, by forming IAPP‐coated SS+GelMA and IAPP‐coated glass+GelMA constructs (**Figure** **8**a). Untreated SS+GelMA and glass+GelMA samples served as controls. We note that for polymeric substrates, PIII can be directly applied to embed radicals within the surface layers, eliminating the need for a deposited coating; as we have previously demonstrated.^[^ ^35^ ^]^ Glass and SS were coated using the same IAPP conditions as Ti. ATR‐FTIR spectra of the IAPP films on both substrates (Figure 8b) showed two broad absorption bands at 3400–3100 cm^−1^ and 1750–1000 cm^−1^, consistent with the spectra observed for IAPP‐coated Ti (Figure 3c), indicating similar surface chemistry. XPS data (Figure 8c) also indicated comparable atomic compositions across all three substrates, highlighting the substrate‐independent nature of the IAPP process.

Figure 8d,e present the ATR‐FTIR spectra of IAPP‐coated glass+GelMA and SS+GelMA before and after SDS washing. The persistence of characteristic GelMA peaks, including the C═O stretch (amide I, ≈1650 cm^−1^) and N–H bending (amide II, ≈1540 cm^−1^), after detergent washing provides evidence of covalent hydrogel attachment on both IAPP‐coated SS and glass. Given that plasma polymerization is a widely established, substrate‐independent process capable of forming coatings on virtually any solid regardless of surface chemistry,^[^ ^56^, ^91^, ^92^ ^]^ these results, together with our data on Ti, collectively demonstrate the universal applicability of the IAPP approach for engineering robust HSHs across a wide spectrum of solid materials. Glass was included in these experiments as an additional model solid substrate, because hydrogel‐coated glass structures are widely used in applications such as cell culture systems,^[^ ^93^ ^]^ optical imaging platforms,^[^ ^94^ ^]^ biosensors,^[^ ^95^ ^]^ and microfluidic platforms, where stable hydrogel anchoring is essential.

Beyond solid‐substrate versatility, we also evaluated the compatibility of the IAPP platform with other hydrogel systems, further demonstrating its effectiveness with UV‐ and chemically crosslinkable hydrogels. In addition to GelMA, two widely used biomedical hydrogels were tested: tyramine‐functionalized poly(vinyl alcohol) (PVA‐Tyr), which can be crosslinked using visible light ^[^ ^96^ ^]^; and chitosan, commonly crosslinked with agents such as glutaraldehyde.^[^ ^97^ ^]^ Figure 8f,g shows the ATR‐FTIR spectra of IAPP‐coated Ti+chitosan and Ti+PVA‐Tyr before and after SDS washing. For chitosan, the retention of saccharide‐related bands at ≈1153 cm^−1^ (C─O─C) and amine signals at ≈1650 cm^−1^^[^ ^98^, ^99^ ^]^ confirms covalent anchoring to the IAPP coating, whereas these peaks disappeared on untreated Ti+chitosan after washing, indicating a physical, non‐covalent interaction. Similarly, IAPP‐coated Ti+PVA‐Tyr retained its hydrogel‐specific peaks at ≈1691 cm^−1^ (C═O) and ≈1315 cm^−1^ (C─O) ^[^ ^100^, ^101^ ^]^ after SDS washing, while these features were absent in untreated Ti+PVA‐Tyr post‐wash controls. Together, these results demonstrate that the IAPP interface supports covalent attachment of hydrogels with varied compositions and crosslinking mechanisms, highlighting its broad applicability in forming stable HSHs across different material systems.

### 3.7 HSH Constructs Support Regenerative and Immune Cell Viability

The cytocompatibility of the HSH constructs was assessed by measuring the viability, metabolic activity, and morphology of stem cells and immune cells cultured on these materials. The study compared untreated Ti, IAPP‐coated Ti, IAPP‐coated Ti+GelMA, and IAPP‐coated Ti+GelMA+Irgacure samples. The IAPP‐coated Ti+GelMA+Irgacure configuration was included to address applications requiring thicker hydrogel coatings. Moreover, incorporating GelMA+Irgacure as a standard hydrogel crosslinking method allows comparison with thin GelMA coatings crosslinked through radicals generated by the plasma polymer layer in this study.^[^ ^102^ ^]^

**Figure 9:**
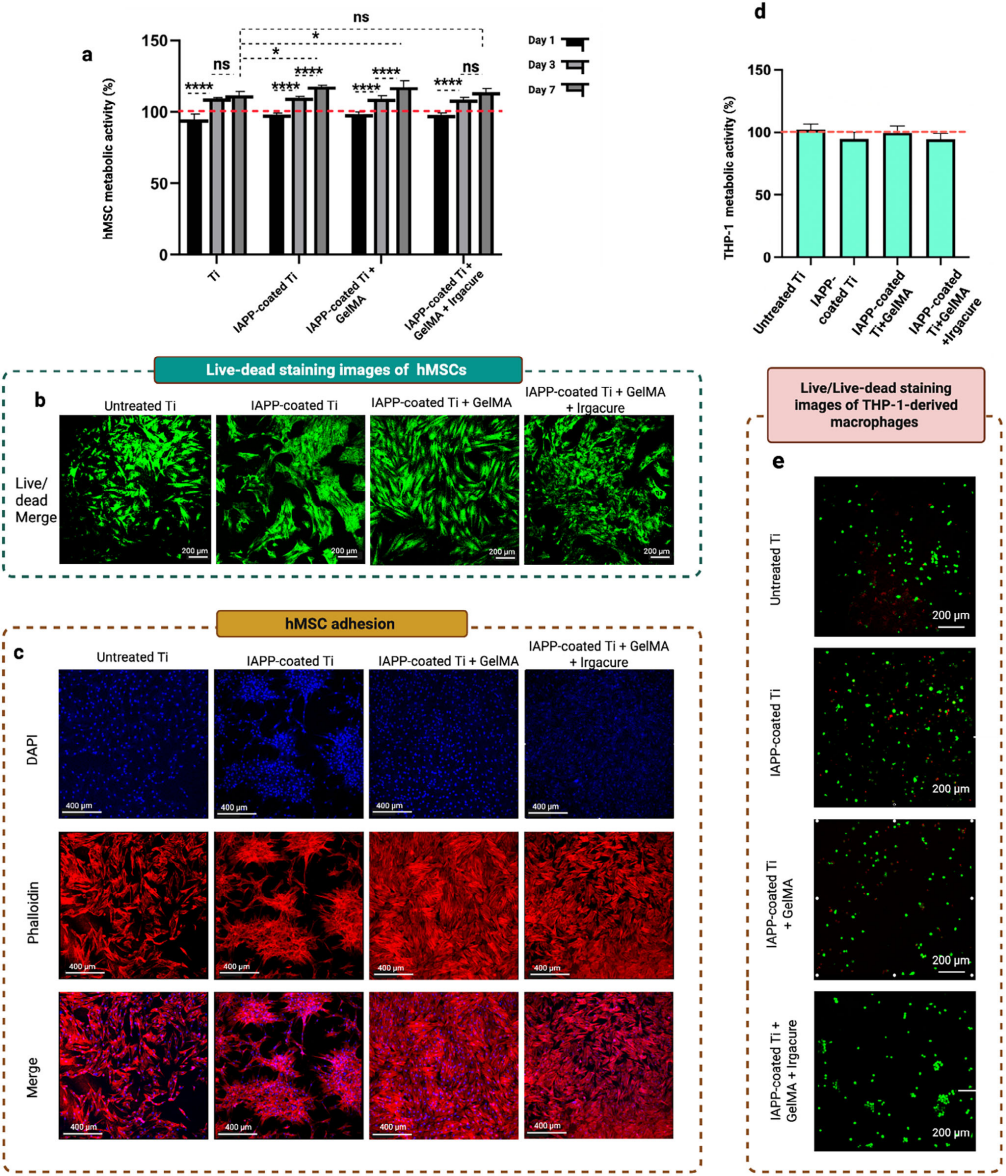
Cytocompatibility of HSH materials. a) Relative abundance of hMSCs on untreated Ti, IAPP‐coated Ti, IAPP‐coated Ti+GelMA, and IAPP‐coated Ti+GelMA+Irgacure samples over 7 days, referenced against cells seeded on tissue culture plastic for 1 day (red dotted line). *n* = 3, ^*^ *p* < 0.05 was considered statistically significant; asterisks denote significance levels: ^*^ *p* < 0.05, ^**^ *p* < 0.01, ^***^ *p* < 0.001, ^***^ *p* < 0.0001. b) Live/dead staining of hMSCs on day 7, showing live cells (green) and dead cells (red). c) Confocal microscopy images of hMSCs stained with DAPI (blue) and Alexa Fluor 488 Phalloidin (red), cultured on various substrates for 7 days.(*n* = 3) d) Abundance of THP‐1‐derived macrophages cultured for 1 day on various substrates. e) Live/dead staining images of THP‐1‐derived macrophages after 1 day of culture, showing live cells (green) and dead cells (red), analyzed using CSLM.

The metabolic activity of hMSCs, as a model for regenerative stem cells, was evaluated using the WST‐8/CCK kit, with results normalized to cells seeded on tissue culture plastic for one day. As illustrated in **Figure** **9**a, cell activity, as a measure of cell abundance, was similar across all samples and comparable to that on tissue culture plastic on day 1 post‐seeding, demonstrating high cell attachment and viability. By days 3 and 7, cell abundance had increased on IAPP‐coated Ti and IAPP‐coated Ti+GelMA compared to the previous time point, indicating that these samples effectively supported cell proliferation over time. Moreover, after 7 days of culture, cell abundance on IAPP‐coated Ti and IAPP‐coated Ti+GelMA was significantly higher than on untreated Ti (control), indicating enhanced proliferation on HSH associated with the IAPP technique.

The cytocompatibility of the GelMA‐based HSHs was further confirmed by live/dead staining of hMSCs on day 7, as shown in Figure 9b. The absence of dead cells (red) on all samples indicates that the cells are viable on these materials, consistent with the cell proliferation shown in Figure 9a. Moreover, cytoskeletal staining of hMSCs after 7 days demonstrated a spindle‐like morphology consistent with well‐adhered cells, as shown in Figure 9c. The hMSCs were also extensively and uniformly distributed on GelMA‐based HSH surfaces. Such consistent cell attachment across the material is likely attributed to the increased surface hydrophilicity and the presence of RGD sequences in the GelMA coating, which facilitate cell‐surface interaction and promote a favorable environment for hMSC attachment.^[^ ^103^, ^104^ ^]^

The cytocompatibility of HSHs toward immune cells was also evaluated by assessing the metabolic activity of THP‐1‐derived macrophages after one day of incubation on the samples. Unlike proliferative cells such as hMSCs, macrophages do not divide after differentiation.^[^ ^105^ ^]^ Instead, they are involved in immune responses, cytokine production, and phagocytosis.^[^ ^106^ ^]^ On this basis, cytotoxicity assessments for THP‐1‐derived macrophages were performed after a short culture duration. On day 1 post‐seeding, cell abundance across all samples was comparable to that of cells seeded on tissue culture plastic controls (Figure 9d). Corresponding live/dead staining images in Figure 9e further support the metabolic activity results from Figure 9d, showing negligible presence of dead cells, confirming the cytocompatibility of the samples.

## 4 HSHs Do Not Elicit a Detectable Inflammatory Response

Biomaterials have the potential to elicit an immune response, due to their recognition by the body as foreign substances.^[^ ^107^ ^]^ When biomaterials are recognized as foreign bodies, the immune system triggers an inflammatory response characterized by the activation of immune cells, cytokine release, and, consequently, the recruitment of additional immune components such as macrophages, T cells, and dendritic cells.^[^ ^108^, ^109^ ^]^ While this response is vital for defense, an excessive or prolonged immune reaction to biomaterials can result in chronic inflammation, fibrosis, encapsulation, degradation, device failure, and delayed healing, compromising their functionality and tissue integration.^[^ ^107^, ^108^, ^110^ ^]^ Thus, in addition to being cytocompatible with immune cells, biomaterials must also avoid triggering an excessive immune reaction.^[^ ^111^, ^112^ ^]^

**Figure 10:**
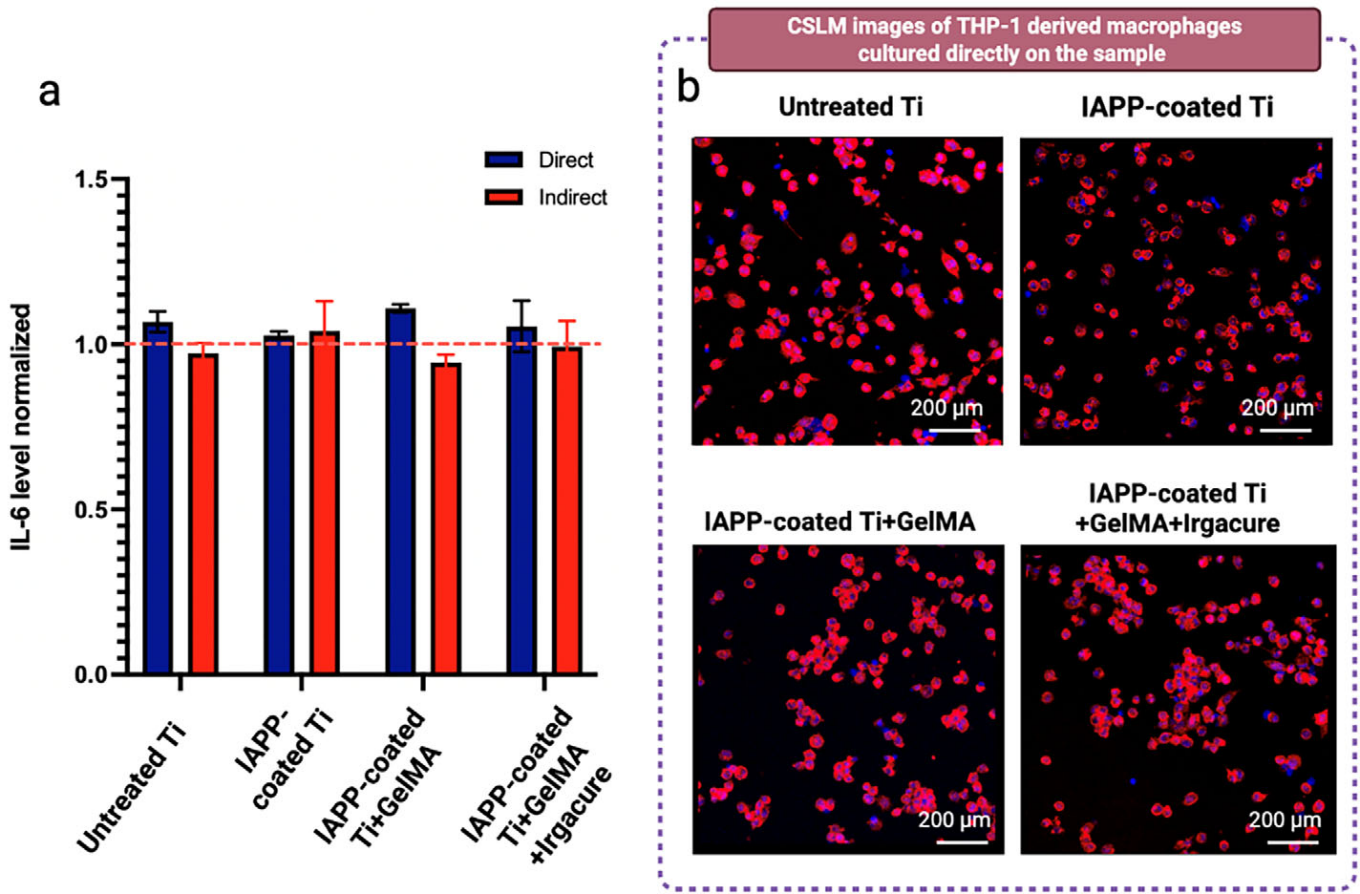
Immune cell responses to HSH materials. a) IL‐6 expression levels in samples where THP‐1 cells were cultured either on the sample surface (direct) or exposed to sample‐incubated media (indirect). b) CSLM images of THP‐1 derived macrophages cultured directly on the sample surfaces for a day and stained for actin.

To assess whether our HSHs trigger an immune reaction, macrophage expression of the pro‐inflammatory cytokine interleukin 6 (IL‐6) was quantified. M0 macrophages were cultured either on HSH constructs or in media incubated with the constructs, to determine immune responses caused by direct contact with the material surface, and those resulting from material degradation. Expression levels of IL‐6, normalized to cells cultured on tissue culture plastic, were similar across all conditions (**Figure** **10**a), demonstrating that neither the surface properties nor the degradation by‐products of the samples induced an inflammatory response.

The absence of inflammatory triggers was further validated by examining the morphology of macrophages cultured on the sample surfaces. Macrophages in their M0 state generally have a rounded or slightly elongated morphology, whereas polarization to the pro‐inflammatory M1 state results in more pronounced elongation, an irregular shape, and the presence of extended pseudopods.^[^ ^113^ ^]^ Confocal images of THP‐1 cells, presented in Figure 10b, showed no detectable changes in cell morphology suggestive of M1 characteristics in any of the samples, further confirming the absence of pro‐inflammatory cues from the materials. Together, these results indicate that IAPP‐based HSHs are immunologically inert, supporting their use in regenerative and implantable biomedical applications.

### 4.1 Bioinstructive Control of Soft Tissue Cell Behavior via HSH Interfaces

Soft tissue integration refers to the stable adhesion and functional incorporation of implants into the surrounding tissue. This process is influenced by cell adhesion, spreading, proliferation, and migration, in response to the implant surface. To determine if soft tissue integration of solid implants can be finely tuned through IAPP‐based surface modification, HSHs were developed using two distinct hydrogels with contrasting properties. GelMA, a bioactive hydrogel containing cell‐binding RGD motifs, was selected to promote cell adhesion and proliferation for enhanced tissue integration.^[^ ^114^, ^115^ ^]^ In contrast, PVA‐Tyr, a bioinert hydrogel, was selected to minimize cell interactions for a low‐fouling surface.^[^ ^38^, ^39^ ^]^ To add another layer of functional control, Tropoelastin (TE), a cell‐instructive extracellular matrix component,^[^ ^116^ ^]^ was variably incorporated into the HSHs to form IAPP‐coated Ti+GelMA+TE and IAPP‐coated Ti+PVA‐Tyr+TE.

**Figure 11:**
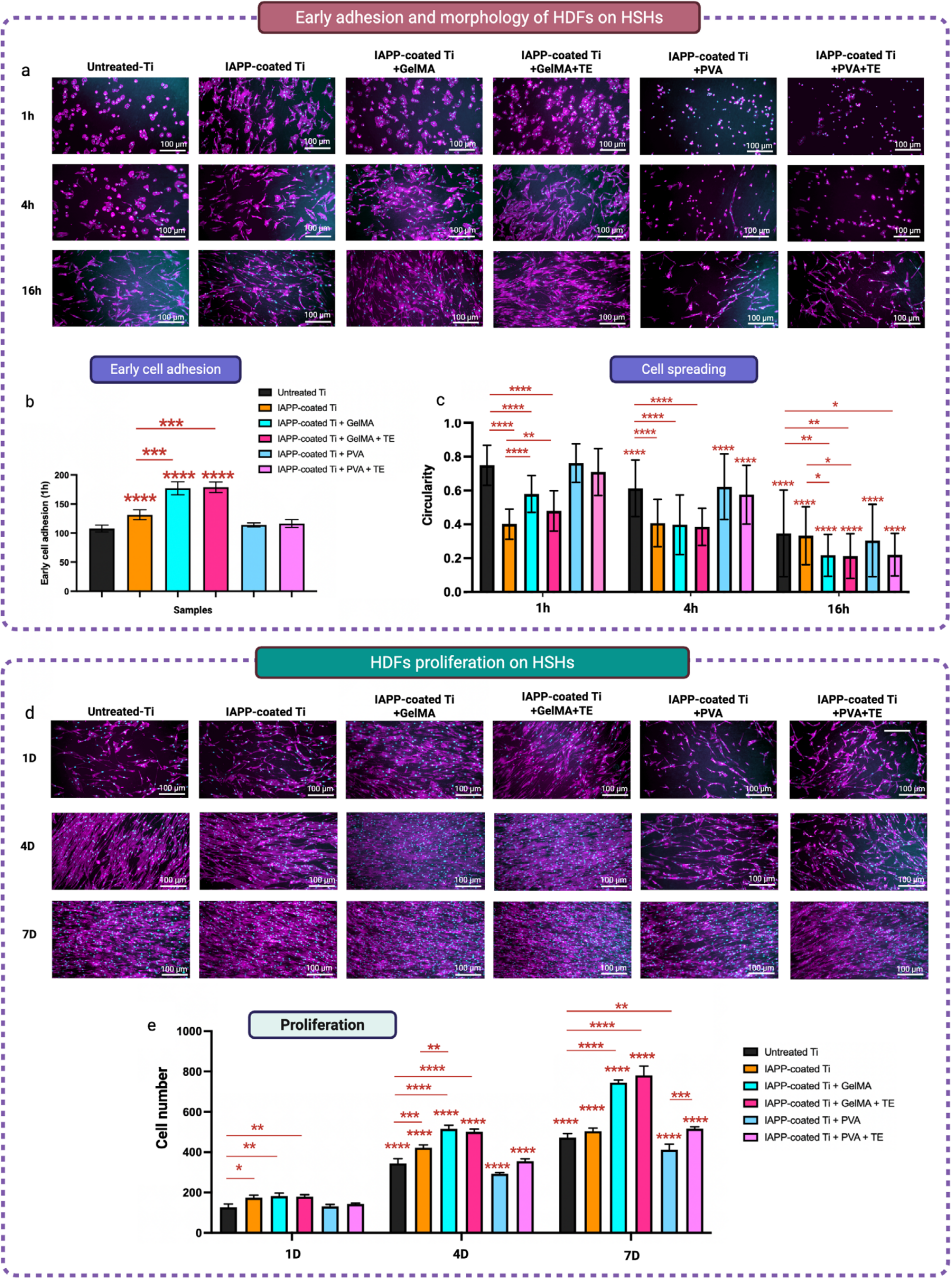
HDF responses to inert or active HSHs. a) Fluorescent images showing cells stained for actin (magenta) and nuclei (cyan) after 1, 4, and 16 h of culture b) Cell numbers on the various surfaces after 1 h of adhesion (*n* = 9). Asterisks placed above columns indicate comparison with the untreated Ti control. c) Cell circularity was assessed at 1, 4, and 16 h. Asterisks directly above the columns indicate differences compared to the same sample at the previous time point (*n* = 90). d) Fluorescent images of cells, and e) cell numbers after 1, 4, and 7 days of culture on various substrates. Asterisks above the columns denote statistically significant differences relative to the same sample at the previous time point. *n* = 9, Asterisks denote significance levels: ^*^ *p* < 0.05, ^**^ *p* < 0.01, ^***^ *p* < 0.001, ^***^ *p* < 0.0001.

As the primary cell type in soft tissues, fibroblasts contribute significantly to tissue integrity, regeneration, and the integration of implanted materials.^[^ ^117^, ^118^, ^119^ ^]^ On this basis, human dermal fibroblast (HDF) adhesion, spreading, and proliferation on HSHs were evaluated (**Figure** **11**). One hour after cell seeding, Ti surfaces coated with IAPP, GelMA, or GelMA+TE exhibited significantly enhanced cell attachment compared to uncoated Ti (Figure 11a,b). Notably, GelMA and GelMA+TE coatings supported a greater number of adherent cells than IAPP, with no difference observed between GelMA and GelMA+TE surfaces. In contrast, PVA‐Tyr‐ and PVA‐Tyr+TE‐coated surfaces showed comparable cell attachment to untreated Ti after 1 h of culture.

Cell spreading profiles on the various substrates were reflective of the cell adhesion patterns. Circularity ^[^ ^120^, ^121^ ^]^ (also referred to as compactness ^[^ ^122^ ^]^) measures the degree to which cell shape resembles a circle. Cell circularity is an indicator of cell spreading, in which a lower circularity value reflects a more spread morphology, while a higher circularity value suggests a more rounded morphology due to limited adhesion and interaction with the substrate.^[^ ^120^, ^123^ ^]^ Cell circularity decreased on all samples during the first 16 h, indicating the ability of HDFs to adhere and spread on these surfaces (Figure 11c).

At the earliest time point of 1 h post‐seeding, HDFs on IAPP‐, GelMA‐, and GelMA+TE‐coated Ti exhibited rapidly decreased circularity values, and hence increased cell spreading, compared to other groups. IAPP‐coated Ti supported higher cell spreading than GelMA or GelMA+TE Ti. In contrast, cells on untreated Ti, PVA‐Tyr, and PVA‐Tyr+TE surfaces exhibited a rounded morphology with the highest circularity values, indicating restricted spreading. After 4 h of culture, a similar trend was observed, with IAPP‐coated Ti, GelMA, and GelMA+TE showing the lowest circularity values, indicating extensive spreading. No significant difference was detected among these three coatings. In contrast, cells on PVA‐Tyr and PVA‐Tyr+TE maintained high circularity, consistent with the 1 h results, indicating limited spreading capability on these surfaces. The addition of TE to the PVA‐Tyr coating did not enhance early cell adhesion and spreading capability over 4 h of culture. After 16 h of culture, HDFs on GelMA and GelMA+TE exhibited the lowest circularity values compared to both untreated Ti and IAPP‐coated Ti, indicating the highest degree of cell spreading. Cells on PVA‐Tyr coating maintained a similar degree of spreading to untreated Ti, whereas cells on PVA‐Tyr+TE showed improved spreading than those on untreated Ti, pointing to the potential of using TE to temporally modulate the biocompatibility of inert hydrogels.

The rapid cell attachment and spreading on IAPP‐coated Ti surfaces after even 1h of culture can be explained by the introduction of functional groups —such as amines, hydroxyls, and carboxylic acids— in the plasma‐polymerized layer, enhancing protein adsorption and integrin binding for improved cell adhesion.^[^ ^124^, ^125^ ^]^ The increased cell attachment and spreading observed on GelMA and GelMA+TE coated Ti samples after 1h of culture, compared to untreated Ti, can be attributed to the presence of cell‐adhesive RGD sequences and amine groups in GelMA, as well as domains in TE that bind to cell‐surface receptors such as integrins and glycosaminoglycans.^[^ ^126^, ^127^, ^128^ ^]^ These sequences can induce cytoskeletal filopodial extensions, which anchor cells, facilitate environmental sensing,^[^ ^129^, ^130^, ^131^ ^]^ and promote integrin clustering and focal adhesion formation essential for mechanotransduction.^[^ ^132^, ^133^ ^]^ The activation of intracellular pathways further enhances cytoskeletal organization, promoting cell spreading.^[^ ^134^ ^]^ The cell attachment and eventual cell spreading observed on PVA‐Tyr surfaces are likely attributed to the hydroxyl (OH) groups in the PVA‐Tyr structure.^[^ ^38^ ^]^ OH groups in PVA‐Tyr may interact with proteins, growth factors, and other components in the serum‐containing cell culture media, facilitating indirect cell interactions.^[^ ^135^, ^136^ ^]^ The enhanced spreading on PVA‐Tyr+TE after 16 h compared to PVA‐Tyr alone is likely due to the incorporation of TE, which introduces cell‐adhesive cues that promote cell‐surface interactions.^[^ ^127^, ^128^, ^137^ ^]^

The influence of active and inert HSH coatings on HDF proliferation over a 7‐day period was evaluated. Fluorescence microscopy images at days 1, 4, and 7 are presented in Figure 11d, and quantified in Figure 11e. Even at day 1 post‐seeding, IAPP, GelMA, and GelMA+TE coatings displayed a higher number of cells compared to untreated Ti (Figure 11e). In contrast, PVA‐Tyr and PVA‐Tyr+TE showed similar cell numbers to untreated Ti, indicating that PVA‐Tyr‐based HSH materials do not enhance cell adhesion after 1 day. These findings are consistent with the relative cell adhesion properties of the samples at the earliest observed timepoint of 1 h, as shown in Figure 11b.

Over 7 days, all samples demonstrated a significant increase in cell numbers compared to day 1, highlighting their pro‐proliferative capacity. GelMA and GelMA+TE surfaces significantly promoted cell proliferation compared to untreated and IAPP surfaces, with no discernible difference between GelMA and GelMA+TE. These results suggest that the inherent bioactivity of GelMA‐based hybrid structures is sufficient to maximally enhance HDF proliferation. In contrast, PVA‐Tyr coatings showed significantly lower cell proliferation compared to untreated Ti, consistent with their inert nature. The addition of TE to PVA‐Tyr enhanced cell proliferation by day 7, although not to the same extent as the GelMA coatings.

Modulating soft tissue integration has critical implications for implant success. For dental implants ^[^ ^138^ ^]^ and cardiovascular devices like stents,^[^ ^139^ ^]^ improving soft tissue integration promotes implant stability, reduces the risk of infection, and enhances overall implant functionality and longevity. Conversely, preventing premature tissue attachment to temporary implants ^[^ ^140^, ^141^ ^]^ or anti‐cancer drug delivery coatings ^[^ ^142^, ^143^ ^]^ is needed for optimal performance and reduced adverse effects. Moreover, incorporation of bioactive matrix cues such as TE can also provide additional tuneability and versatility to these cell‐instructive surface coatings.

The IAPP‐enabled platform offers a versatile and programmable interface for engineering HSH systems with tunable biological responses, paving the way for application‐specific control of implant–tissue integration. The versatility of such HSH constructs opens pathways to a wide range of applications, including soft tissue‐integrating implants, biosensing platforms, advanced 3D cell culture systems, and beyond.

## 5 Conclusion

Creating robust, long‐lasting hydrogel–solid interfaces without relying on chemical linkers or initiators remains a critical challenge for the translation of hydrogel‐based materials into biomedical applications. In this study, we developed a dry, scalable strategy for fabricating HSH materials using radical‐rich IAPP coatings. This reagent‐free approach enabled covalent attachment and spontaneous crosslinking of hydrogels on chemically diverse substrates, including titanium, stainless steel, and glass. The long‐lived radicals embedded within the IAPP layer underpinned both interfacial bonding and crosslinking mechanisms, as demonstrated by EPR‐based radical quenching and FTIR spectral changes in the hydrogel structure. The hydrogel coatings remained stable for up to two months in aqueous media at 37 °C, and showed strong interfacial adhesion (Γ_t_ > 251 ± 12 N m^−1^) confirmed by peel testing. The platform supported a range of hydrogel chemistries, including GelMA, chitosan, and PVA‐Tyr. GelMA‐based HSHs supported viability and normal morphology of human MSCs and THP‐1‐derived macrophages, with no signs of inflammatory activation. Soft tissue responses were further modulated using bioactive and bioinert hydrogels, with fibroblasts showing enhanced adhesion and proliferation on GelMA‐based constructs. As an additive‐free and initiator‐free approach, this method represents a high‐quality‐by‐design strategy, offering precise, clean, and scalable surface functionalization. Collectively, these findings establish a universal and reagent‐free interface engineering strategy, underpinned by solid‐state radical functionality, with broad utility across biomedical coatings, implantable devices, and soft–hard tissue integration. In future works, the substrate‐independent nature of IAPP coatings can enable extension of this platform to other technologically relevant solids, such as elastomeric materials used in wearable devices, soft robotics, and implantable electronics, where hydrogel–elastomer adhesion remains a major bottleneck.

## Conflict of Interest

The authors declare no conflict of interest.

## Supporting information



Supporting Information

## Data Availability

The data that support the findings of this study are available from the corresponding author upon reasonable request.
