# Supplementary material for: Radical‐Mediated, Substrate‐Independent Fabrication of Hybrid Solid–Hydrogel Materials With Tunable Crosslinking: An Initiator‐ and Crosslinker‐Free Approach
Source: Adv Sci (Weinh). 2026 Jan 21;13(27):e16300. doi: 10.1002/advs.202516300 (PMC13170253; doi:10.1002/advs.202516300)
Supplement: Supplementary file 1 — Supporting Information [file ADVS-13-e16300-s001.docx]

Supplementary Information

**Radical-mediated, substrate-independent fabrication of hybrid solid–hydrogel materials with tunable crosslinking: An initiator- and crosslinker-free approach**

Ghazal Shineh ^a,b,c^, Azin Khodaei ^d^, Pardis Keikhosravani ^d^, Masoud Zhianmanesh ^a,b^, Yiyun Xia^b^, Aaron Glimour ^a,c^, Sina Naficy ^e^, Andrea Guzina ^a^, Daniella Bianchi ^a^, Khoon s Lim ^c, f^, Fariba Dehghani ^e^, Saber Amin Yavari ^d,g^ , Marcela Bilek ^a,c,h,i^, Giselle C. Yeo ^c,h,j^, Behnam Akhavan ^a,b,h,I,k^*

*^a^ School of Biomedical Engineering, University of Sydney, Sydney, New South Wales 2006, Australia*

*^b^ School of Engineering, University of Newcastle, Callaghan, NSW 2308, Australia*

*^c^ Charles Perkins Centre, University of Sydney, NSW 2006, Australia*

*^d^ Department of Orthopedics, University Medical Center Utrecht, Utrecht 3508GA, the Netherlands*

*^e^ School of Chemical and Biomolecular Engineering, The University of Sydney, Darlington, NSW 2008, Australia*

*^f^ School of Medical Sciences, The University of Sydney, Darlington, NSW 2008, Australia*

*^g^ Regenerative Medicine Utrecht, Utrecht University, Utrecht, the Netherlands*

*^h^ The University of Sydney Nano Institute, University of Sydney, Sydney NSW 2006, Australia*

*^i^ School of Physics, University of Sydney, Sydney NSW 2006, Australia*

*^j^ School of Life and Environmental Sciences, University of Sydney, NSW 2006, Australia*

*^k^ Hunter Medical Research Institute (HMRI), New Lambton Heights, NSW 2305, Australia*

** Corresponding author:*

[*Behnam.Akhavan@Newcastle.edu.au*](mailto:Behnam.Akhavan@Newcastle.edu.au)*;* [*behnam.akhavan@Sydney.edu.au*](mailto:behnam.akhavan@Sydney.edu.au)

# **1. Materials and Experimental Sections**

## **1.1. Materials**

Low-density polyethylene (LDPE) sheets, 0.2 mm in thickness, were obtained from Goodfellow (UK).

**2. Single-LAP shear test**

Single-lap shear testing was used to evaluate the adhesion of GelMA hydrogels to LDPE in both dried and hydrated states. Untreated and IAPP-modified low-density polyethylene (LDPE) sheets were cut into 1 × 5 cm strips. A GelMA precursor solution (10% w/v with 1% w/v Irgacure 2959) was prepared at 40–50 °C until fully dissolved. Each LDPE strip was placed on a flat glass, and a silicone mold (2 × 1 cm, 1 mm thickness) was positioned on the substrate. Next, 180 µL of the GelMA pre-gel solution was dispensed into the mold on the first LDPE strip (for treated samples, they were facing upward), after which a second LDPE strip (for treated, they were facing downward) was placed over the solution to create a sandwich structure. The assembled samples were transferred to a UV chamber and exposed to 365 nm light for 1 hour to achieve complete photopolymerization. This arrangement produced a GelMA layer roughly 1 mm thick positioned between the two LDPE sheets as we showed in our previous study [1]. Following crosslinking, the constructs were removed from the mold and left to dry under ambient conditions for 24 h.

Dried IAPP–LDPE constructs were then separated into two test groups. For hydrated testing, samples were submerged in Milli-Q water for 4 h prior to analysis. For dehydrated testing, samples remained fully dry until loading. Untreated LDPE controls were directly immersed in Milli-Q water before testing. Five specimens were prepared for each condition.

Lap-shear measurements were carried out using a Shimadzu AGS-X universal testing system fitted with a 500 N load cell. One LDPE strip was held stationary while the opposing strip was pulled in shear, parallel to the hydrogel–substrate interface, at a crosshead speed of 10 mm min⁻¹. Force–displacement data were converted to stress–displacement by dividing the recorded force by the bonded surface area.

**
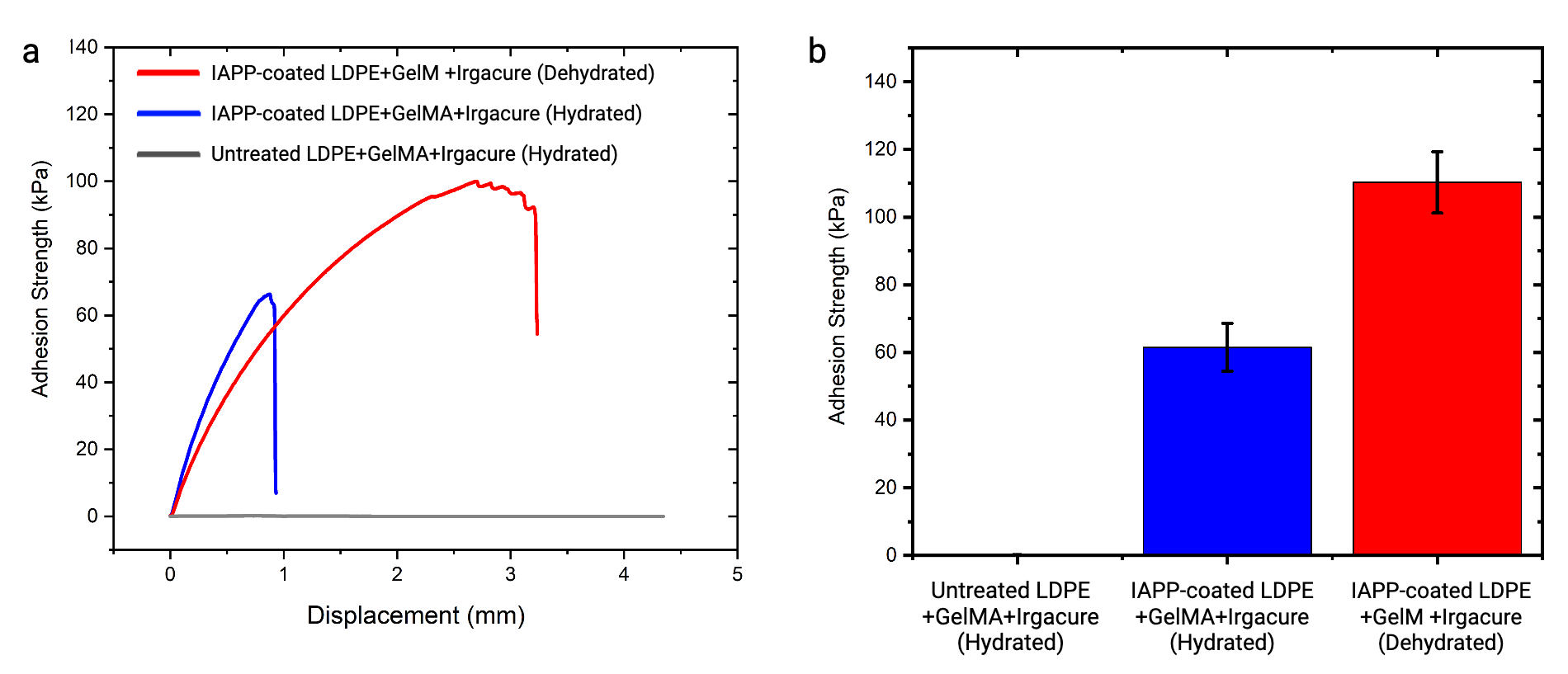
**

**Figure S1.** Lap-shear test results for GelMA–LDPE hybrid constructs. a) Adhesion strength–displacement curve for hydrated and dehydrated IAPP–coated LDPE+GelMA and hydrated untreated LDPE+GelMA+Irgacure, and b) Corresponding adhesion strength, demonstrating negligible adhesion strength on untreated LDPE and robust adhesion on IAPP-activated LDPE.

References

1. Zhianmanesh M, Khodaei A, Crago M, Lotz O, Naficy S, Dehghani F, et al. Universal Method for Covalent Attachment of Hydrogels to Diverse Polymeric Surfaces for Biomedical Applications. Advanced Materials. 2025:e03524.
